# Estimating returns to education using the genetic lottery

**DOI:** 10.1073/pnas.2537049123

**Published:** 2026-04-08

**Authors:** Tarjei Widding-Havneraas, Perline A. Demange, Henrik Daae Zachrisson, Nicolai Borgen, Eivind Ystrom, Felix Elwert

**Affiliations:** ^a^Centre for Research on Equality in Education, University of Oslo, Oslo 0318, Norway; ^b^Department of Special Needs Education, University of Oslo, Oslo 0318, Norway; ^c^PROMENTA Research Center, Department of Psychology, University of Oslo, Oslo 0317, Norway; ^d^Department of Child Health and Development, Norwegian Institute of Public Health, Oslo 0473, Norway; ^e^Department of Sociology, University of Wisconsin-Madison, Madison, WI 53705; ^f^Department of Biostatistics and Medical Informatics, University of Wisconsin-Madison School of Medicine and Public Health, Madison, WI 53705; ^g^Department of Population Health Sciences, University of Wisconsin-Madison School of Medicine and Public Health, Madison, WI 53705

**Keywords:** returns to education, Mendelian randomization, quasi-experiment, The Norwegian Mother, Father and Child Cohort Study (MoBa), The Norwegian Twin Registry (NTR)

## Abstract

Does more schooling cause higher lifetime earnings? Social scientists have long sought to determine the economic returns to schooling given its importance to individual life chances and public policy. Prior estimates are limited by unobserved confounding in observational studies and the focus of popular quasi-experimental studies on increases in schooling at only one particular age. Genotyped data offer an opportunity to address unobserved confounding and to estimate the returns to an additional year of schooling at any age by using quasi-randomly assigned genetic variants related to education as instrumental variables [Mendelian randomization (MR)]. We analyze comprehensive Norwegian population registries with career-long earnings data and genotyped data from the Norwegian Mother, Father and Child Cohort Study (MoBa). We employ three identification strategies for triangulation: ordinary least squares (OLS) with covariate-adjustment, sibling and twin fixed-effects models, and MR. Estimated returns to schooling are 8.0% in MR (N = 109,800) and 6.3% in sibling-MR (N = 18,666). Extensive sensitivity analyses suggest that MR results are robust even to large potential violations of MR assumptions, including pleiotropy. MR estimates somewhat higher returns than OLS for the full population (5.9%, N = 1,255,604) and fixed-effects models for siblings (5.3%, N = 966,976) or monozygotic twins (3.2%, N = 2,630). The estimated internal rate of return to schooling exceeds opportunity costs of education as proxied by the market interest rate. The lifetime returns to schooling are positive and substantial across all models.

## Significance

The extent to which education increases earnings is widely debated. We use genetic variation linked to schooling as a natural experiment to estimate the causal effect of an additional year of education on earnings. We find that an extra year of schooling increases earnings by about 8%, which is larger than estimates from conventional statistical and sibling-comparison models. These gains exceed the costs of education, implying that education pays off. Our study demonstrates how genetic data can help answer longstanding questions in the social sciences.

The magnitude of economic returns to schooling is central in multiple domains, including private investment decisions (1), education policy (2), cost–benefit analysis (3), and economic forecasting (4). Yet, despite decades of rigorous research, the extent to which education causally impacts earnings remains uncertain (3, 5).

Most research on the returns to schooling estimates Mincer-type equations in observational studies, using ordinary least squares (OLS) to measure the percentage change in earnings associated with each additional year of schooling (2, 3, 6–11). In high-income countries, the economic returns to one additional year of schooling are estimated to be 8.2% on average (2). Unfortunately, estimates from observational studies are vulnerable to bias from unobserved confounding.

Two dominant empirical strategies have been used to address unobserved confounding: family-based designs and quasi-experiments (12). Family-based designs can account for family-level environmental and genetic confounding by comparing schooling and earnings within siblings, and/or monozygotic (MZ) and dizygotic (DZ) twins (13, 14). Such designs tend to estimate lower returns than OLS but are still vulnerable to bias from unobserved confounding at the individual level (e.g., nonshared environments) (5, 12, 13, 15–17).

The most credible estimates to date stem from quasi-experimental, school reform-based instrumental variables (IV) studies that exploit plausibly exogenous changes in the minimum school-leaving age. Many such studies estimate higher returns to schooling (9 to 15%) (5, 18) than OLS, but recent analyses suggest near-zero returns (19–21). School reform-based studies evaluate specific reforms that raise schooling by 1 y at a particular age, however, which limits their generalizability to increases in schooling at other ages (5).

We address these limitations by exploiting the genetic lottery at birth as a quasi-experiment. During meiosis, genetic variants related to educational attainment (EA) are randomly allocated to offspring, creating random variation in the predisposition for schooling among siblings. Mendelian randomization (MR), a form of IV analysis, leverages this random allocation as an exogenous shock to estimate the causal returns to schooling (22, 23). Specifically, we aggregate 335 independent single-nucleotides polymorphisms (SNPs) that were strongly associated with EA in the international EA4 genome-wide association study (GWAS) and their weights (24) into a polygenic index (22, 23), which we use as a polygenic instrumental variable for EA (PIV^EA^, see *Materials and Methods*). Conventional MR exploits all variation in genetic variants between individuals. Sibling-MR more specifically exploits genetic variation between full siblings (22, 25). Under assumptions detailed below, MR designs identify average causal returns to schooling for individuals who complete additional schooling due to their genetic predisposition (compliers) (26, 27).

While two previous studies have used MR to estimate the effect of education on income using UK Biobank data (28, 29), these analyses have at least five limitations. First, they rely on self-reported rather than administratively ascertained income and education in a sample with selective participation, which may bias estimates (30). Second, they use household income rather than the individual labor market earnings conventionally used in the returns to schooling literature. Third, their sibling-MR models are underpowered, resulting in imprecise and statistically nonsignificant estimates. Fourth, the absence of Mincer-type coefficients prevents comparisons to prior estimates (31, 32). Fifth, they estimate average returns across a wide age range (40 to 69 y) (33), which may incur life-cycle bias as the returns to schooling vary across age (7).

Our study contributes in five ways. First, we estimate the economic returns to schooling with well-powered MR and sibling-MR using nationally representative Norwegian registry data and genetic data from the Norwegian Child, Mother, and Father Cohort Study (MoBa) (34).

Second, we triangulate estimates for the returns to schooling across multiple identification strategies that rely on different assumptions: OLS with covariate-adjustment, family-based designs with sibling and twin fixed-effects, and MR. Collectively, these results provide insights into potential bias sources (35). We elucidate differences in the returns to schooling estimated by OLS and MR using an MR-OLS decomposition method (36).

Third, we conduct extensive analyses to assess the key assumptions underlying MR. To be a valid IV, the PIV^EA^ must (A1) be strongly associated with EA (relevance), (A2) share no common causes with the outcome (independence), (A3) only impact earnings through EA (exclusion), and (A4), under heterogeneous effects, only associate with EA in one direction (monotonicity) (22, 23, 26). We test the relevance assumption (A1) using weak-instrument diagnostics (37). We address possible violations of the independence assumption (A2) (e.g., due to population stratification, assortative mating, or dynastic effects), first, by adjusting for key covariates (e.g., parental education and earnings) in MR analyses in our full population sample; second, by demonstrating balance on observed covariates (38); and, third, by estimating well-powered sibling-MR that adjusts for family-level unobserved confounding (22, 25, 28, 29). We address possible violations of the exclusion assumption (A3) (e.g., due to pleiotropic direct effects, whereby the genetic variants in the PIV^EA^ might impact earnings directly through pathways other than EA) in multiple ways. First, all of our MR analyses use only strongly associated genetic variants to maximize relevance and minimize potential pleiotropic variants (39, 40); second, we test for pleiotropy using an MR-Egger intercept test (23); third, we employ multiple pleiotropy-robust estimators, including MR-Egger, MR-Median, MR-Mode, and MR-Corge (22, 23, 40); fourth, we conduct a formal sensitivity analysis that examines the robustness of our estimates to a range of potential pleiotropic direct effects (41). We assess monotonicity (A4) by inspecting covariate-specific weights in the MR-OLS decomposition (36).

Fourth, we address the concern that our main PIV^EA^, which is derived from the international EA4 GWAS (24), may not be portable to Norway by constructing our own family-based GWAS (FGWAS) (42–44) using an exclusively Norwegian discovery sample of full siblings. We then implement a Norway-only MR model on an unrelated Norwegian estimation sample.

Fifth, we address potential life-cycle bias (7) by analyzing long earnings and education panel data and estimating both averaged lifetime returns to schooling and age-specific profiles across the life-cycle.

Our Norwegian setting is characterized by a small open economy, low economic inequality (45, 46), and a universal welfare state with practically free education. However, wealth inequality is comparatively high (47), child poverty and education gaps between rich and poor have increased (48, 49), and economic mobility among poor families has declined in recent decades (45), underscoring the need for stronger causal evidence about the role of education for earnings (50).

Taken together, our study contributes insights into returns to education using genotyped data, compares estimates from the MR design to other established designs, and offers a comprehensive assessment of the validity of the MR design in the context of a universal welfare state.

## Results

**Fig. 1.**
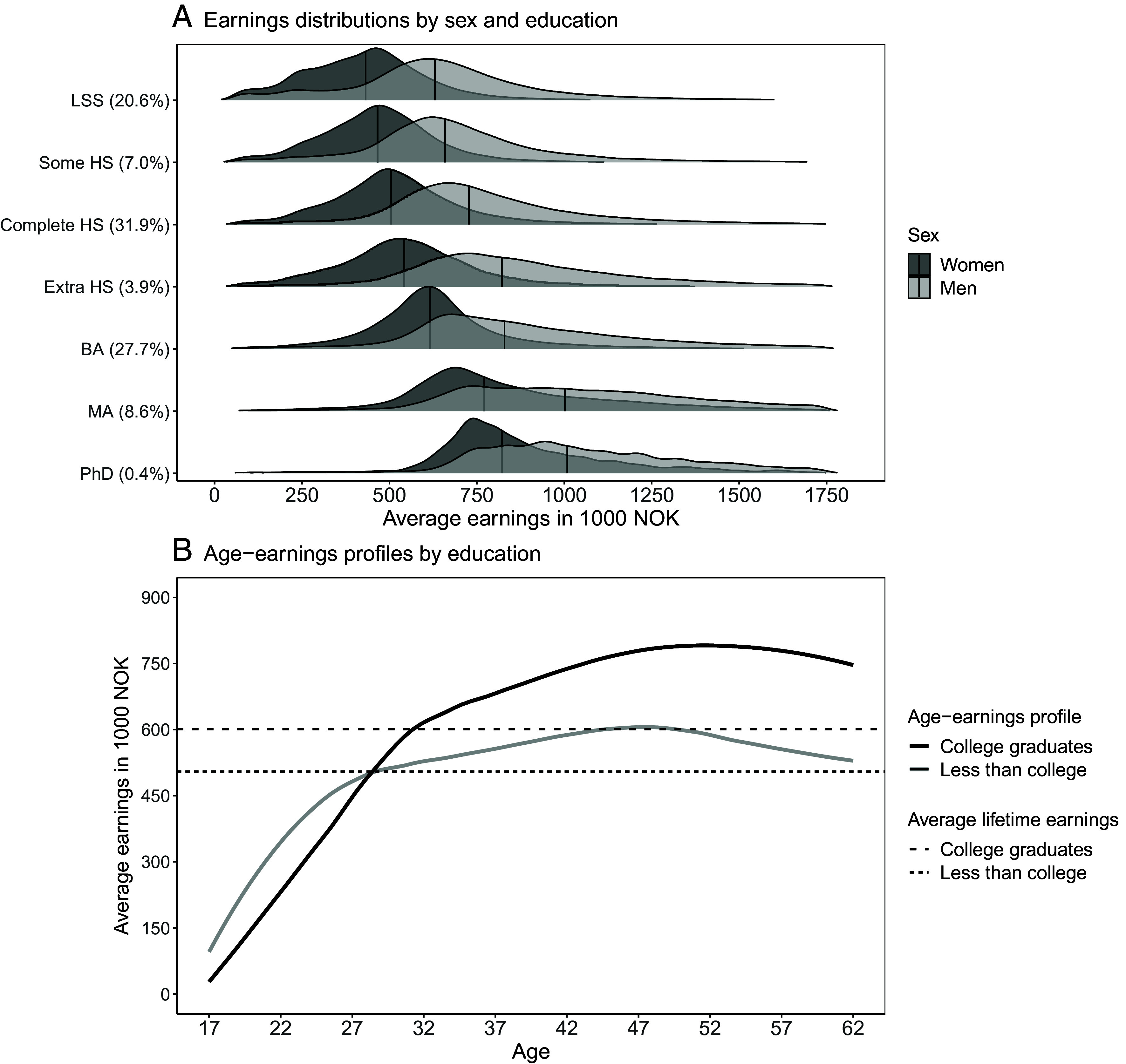
Earnings by educational attainment and age. All individuals born in Norway between 1959 and 1982 (N = 1,255,604) (see *SI Appendix,* Fig. S1 for minor exclusions). Panel *A* shows the distributions of annual earnings (averaged across ages 34 to 40) by sex and levels of final schooling (at age 33) for earnings percentiles 2-98, with medians marked by vertical lines. The percentage of individuals for each educational level is reported in parentheses. LSS = lower secondary school; HS = high school; BA = bachelor’s degree; MA = master’s and professional degrees; PhD = philosophiae doctor. Panel *B* shows loess-smoothed earnings profiles from age 17 to 62 by college education (by age 33), and mean lifetime earnings for college-educated (dashed line) and non-college-educated (short-dashed line) individuals as horizontal lines. Earnings are reported in 1,000s of NOK, wage-inflation adjusted to 2022 levels (USD/NOK ≈ 9.6).

We analyze registry data for all individuals born in Norway between 1959 and 1982 (N = 1,255,604, details in *Materials and Methods*). Fig. 1 presents descriptive results from our full population sample. Panel 1*A* shows that higher levels of schooling are associated with higher earnings. Overall, median annual earnings (averaged over ages 34 to 40) were Norwegian kroners (NOK) 638,368 (IQR = 321,852; USD 66,497, IQR = 33,526). Men had higher earnings [median = NOK 741,004 (IQR = 344,622)] than women [median = NOK 548,805 (IQR = 245,634)]. Panel 1*B* shows the well-known downward-concave age-earnings profiles for college and non-college-educated individuals (7). College graduates forego early-career earnings but experience a steeper earnings increase, surpassing non-college-educated individuals by age 27, thus obtaining overall higher lifetime earnings. Descriptive statistics for all samples are presented in *SI Appendix,* Table S1.

**Fig. 2.**
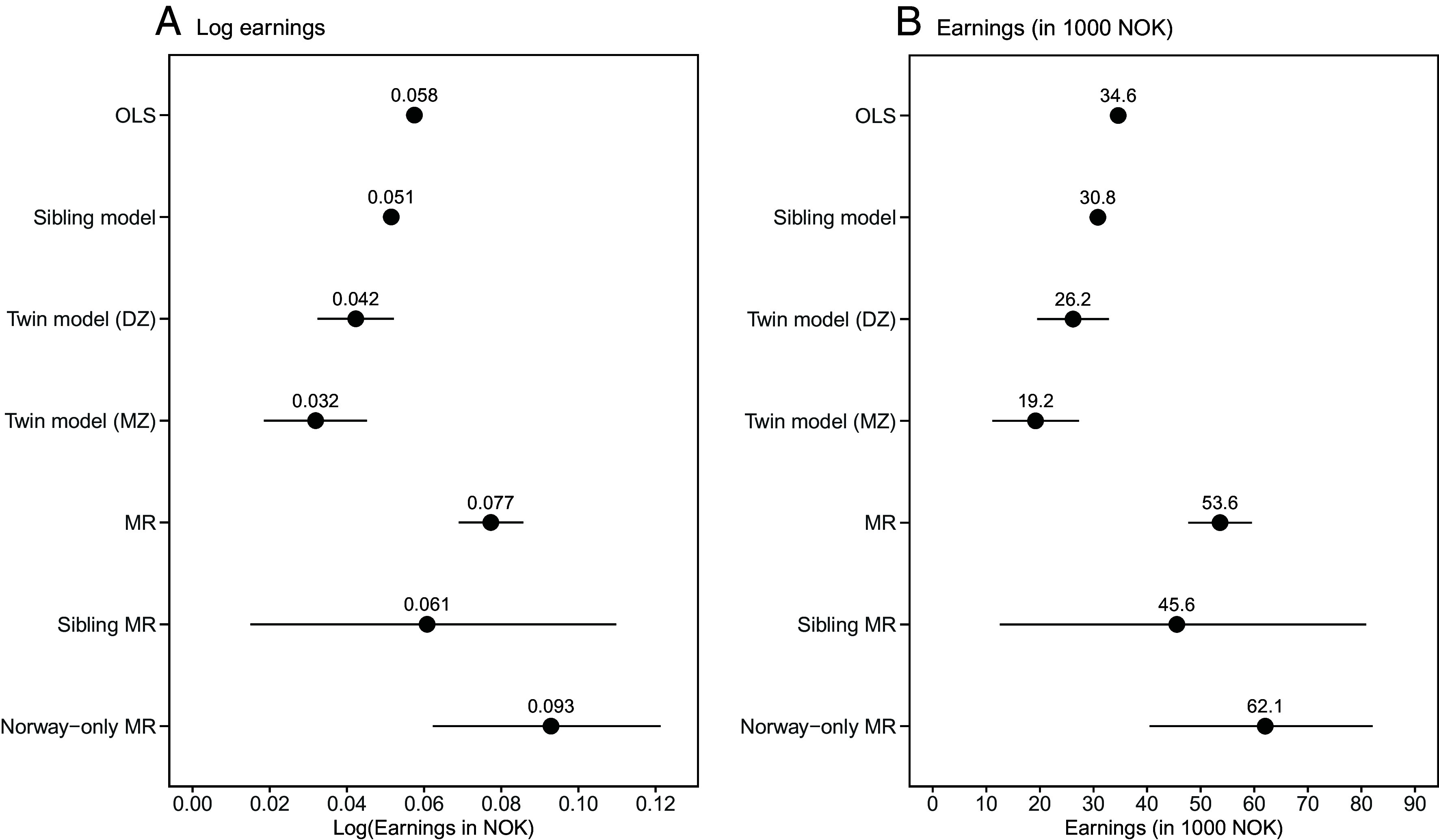
Returns to schooling estimates from OLS, sibling fixed-effects, twin fixed-effects, MR, and sibling-MR models. We report point estimates and 95% CI from the following models and samples (all using birth cohorts 1959-1982; final years of schooling by age 33; earnings averaging the top-three earnings years across ages 34-40): OLS (full population sample: N = 1,255,604), sibling fixed-effects (sibling sample: N = 966,976), twin fixed-effects (DZ) (DZ twin sample: N = 3,219), twin fixed-effects (MZ) (MZ twin sample: N = 2,630), MR (MoBa genotyped sample: N = 109,800), sibling-MR (MoBa genotyped sibling sample: N = 18,666), and Norway-only MR (MoBa genotyped estimation sample excluding individuals in the MoBa within-sibling FGWAS discovery sample: N = 89,179). All models adjusted for covariates and estimated separately for log earnings (Panel A) and absolute earnings (Panel B). SE clustered by family and birth cohort in sibling and twin fixed-effects and sibling-MR models, and by birth cohort otherwise. We report Anderson-Rubin 95% CI for MR, sibling-MR, and Norway-only MR models. OLS = Ordinary least squares; DZ = dizygotic; MZ = monozygotic; FE = Fixed-effects; MR = Mendelian randomization; MoBa = Norwegian Mother, Father, and Child Cohort Study. Earnings reported in 1,000s of NOK wage-inflation adjusted to 2022 levels (USD/NOK ≈ 9.6).

Fig. 2 presents estimates for the returns to schooling from seven models that successively address key threats to validity (estimated separately for log earnings and absolute earnings, detailed results in *SI Appendix,* Table S2). Labor market earnings were the mean of the top-three earnings years between ages 34 and 40. Years of schooling were the highest educational attainment by age 33. All analyses were adjusted for covariates, including sex, parents’ income and education, number of children in the family, birth year, birth order, and parents’ age at birth (sibling and twin fixed-effects models and sibling-MR do not adjust for covariates that are constant between siblings) (*SI Appendix*, sections 1.3 and 2).

First, our OLS estimate from the full population sample provides an observational benchmark. One additional year of schooling was associated with 0.058 log points (95% CI = 0.056-0.059), i.e., 5.9%, higher earnings (Fig. 2*A*). This corresponds to additional earnings of NOK 34,617 (95% CI = 34,087-35,148; USD 3,606, 95% CI = 3,551-3,661) on average (Fig. 2*B*). OLS estimates are vulnerable to all forms of unobserved confounding.

Second, our sibling fixed-effects model, which adjusts for shared, family-level, unobserved confounding by using within-family variation only, gives a lower estimate of 0.051 log points (95% CI = 0.051-0.052), i.e., 5.3%, compared to OLS. This corresponds to additional earnings of NOK 30,823 (95% CI = 30,511-31,139). Third, the DZ twin fixed-effects model, which additionally adjusts for unobserved confounding due to temporal changes in family and prenatal environments, produced a lower yet less precise estimate of 0.042 log points (95% CI = 0.032-0.052), i.e., 4.3%, compared to our sibling model. This corresponds to additional earnings of NOK 26,194 (95% CI = 19,510-32,879). Fourth, the MZ twin fixed-effects model, which additionally accounts for unobserved confounding from the full genome by comparing genetically identical twins, provided the lowest estimate of 0.032 log points (95% CI = 0.018-0.045), i.e., 3.3%, but also had lower precision relative to our sibling model. This corresponds to additional earnings of NOK 19,196 (95% CI = 11,113-27,280).

Fifth, the MR model leverages variation in genetic predisposition for schooling and is robust to all unobserved confounding under assumptions A1-A4 (above). The two-stage least squares (2SLS) MR estimate in the full genotyped MoBa sample provided a higher estimate of 0.077 log points [Anderson-Rubin (AR) 95% CI = 0.069-0.086], i.e., 8.0%, than OLS, sibling, and twin models. This corresponds to additional earnings of NOK 53,622 (AR 95% CI = 47,701-59,544).

Sixth, the sibling-MR model strengthens the credibility of the MR independence assumption (A2) by adjusting for family-level unobserved confounding through exploiting only within-family variation in individuals’ genetic predisposition for schooling (22, 25, 28, 29). The sibling-MR produced a slightly lower estimate of 0.061 log points (AR 95% CI = 0.015-0.110), i.e., 6.3%, than MR, yet still higher than OLS and substantially less precise. This corresponds to additional earnings of NOK 45,560 (AR 95% CI = 12,546-80,891).

Seventh, the Norway-only MR model additionally addresses portability concerns by using the PIV^EA^ constructed from our Norwegian within-sibling FGWAS. The Norway-only MR estimate was 0.093 log points (AR 95% CI = 0.062-0.121), i.e., 9.7%, and thus somewhat higher than the MR estimate from our full genotyped sample and less precise. This corresponds to additional earnings of NOK 62,054 (AR 95% CI = 40,493-82,103).

In sum, the estimated returns to schooling were positive and statistically significant across all models.

### Life-Cycle Analyses.

**Fig. 3.**
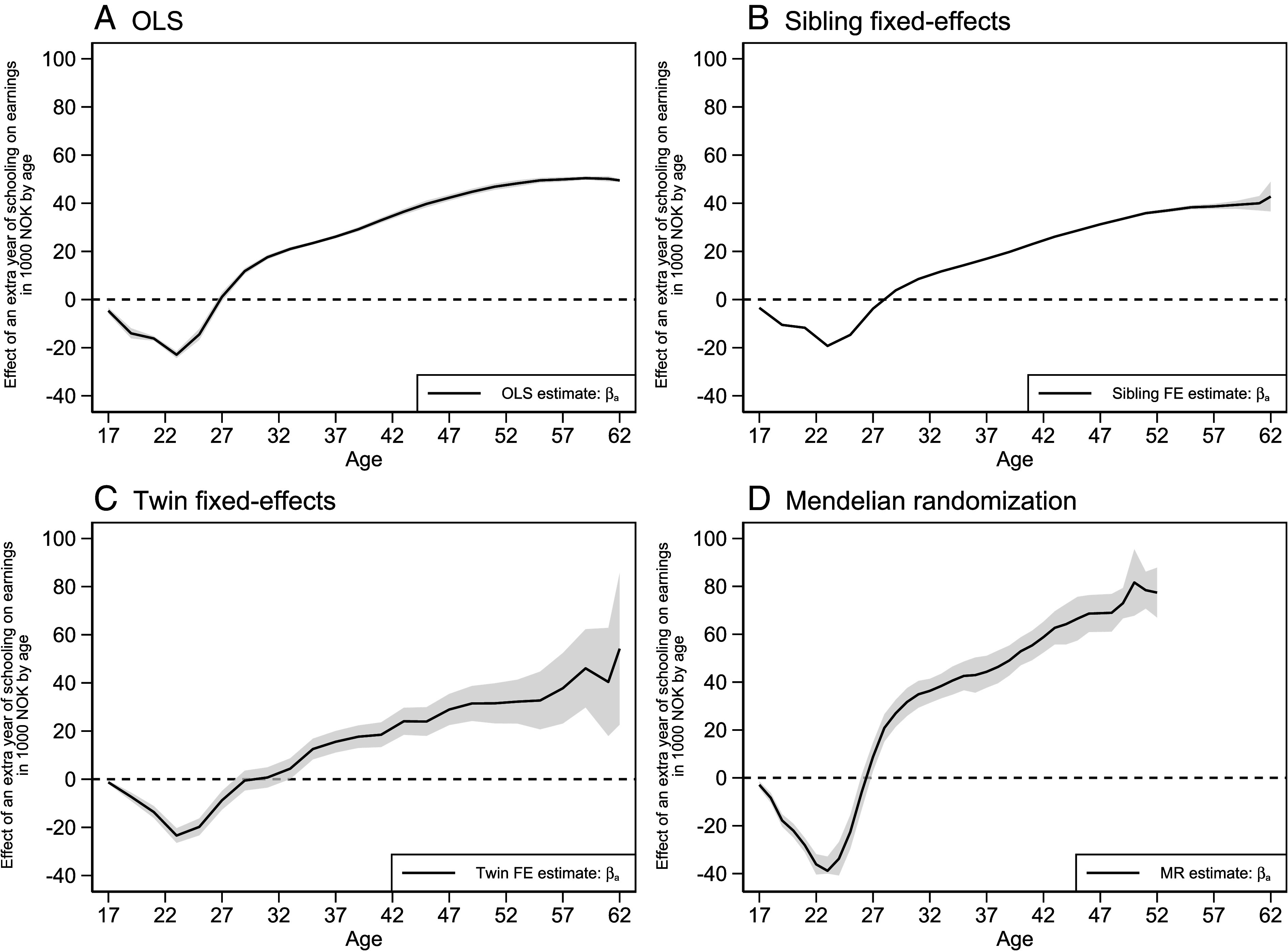
Estimates of age-specific returns to schooling. We report point estimates and 95% CI from: (*A*) OLS, (*B*) sibling fixed-effects, (*C*) twin fixed-effects, and (*D*) MR models (all using birth cohorts 1959-1982). All models are estimated on the same samples as corresponding models in Fig. 2 with different age restrictions and following the methodology of Bhuller, Mogstad, and Salvanes (7) (*SI Appendix*, section 3.5). SE are clustered by birth cohort and childhood municipality. 95% CI represented by shaded areas. OLS = Ordinary least squares; FE = Fixed-effects; MR = Mendelian randomization. Earnings reported in 1,000s of NOK, wage-inflation adjusted to 2022 levels (USD/NOK ≈ 9.6).

Fig. 3 presents age-specific returns to schooling across the life-cycle following Bhuller, Mogstad, and Salvanes (7) (*SI Appendix*, section 3.5). This approach allows us to examine how returns develop over individuals’ working careers [ages 17-62 (17-52 for MR)]. Across all estimation approaches, an additional year of schooling had negative returns until the late 20s before becoming positive and increasing in size over the work career (*SI Appendix,* Table S9, Panel A).

Additionally, we present estimated lifetime returns to an additional year of schooling as the undiscounted average of age-specific returns (*SI Appendix,* Table S9, Panel B). As expected, lifetime estimates were lower than the main specifications for prime-age individuals (Fig. 2, using earnings from ages 34 to 40) due to the inclusion of negative early-career returns. The estimated lifetime returns were NOK 13,411 (95% CI = 11,347-15,474), i.e., 2.8%, in OLS, NOK 8,403 (95% CI = 8,183-8,623), i.e., 1.7%, in the sibling fixed-effects model, NOK 5,542 (95% CI = 2,685-8,399), i.e., 1.1%, in the twin fixed-effects model, and NOK 21,544 (95% CI = 17,180-25,909), i.e., 4.3%, in MR, respectively (*SI Appendix,* Table S9).

We estimate the internal rate of return (IRR) to schooling, found by equating life-cycle costs (e.g., forgone earnings while in school) and life-cycle benefits (higher postschooling earnings) when both are converted to their present value (5, 7). Education is profitable if the IRR exceeds opportunity costs, as measured by the market interest rate. Consistent with prior work (7), the estimated IRR (*SI Appendix,* Table S9, Panel C) greatly exceeded the real market interest rate [2.3% (51)] in all specifications [OLS: 10.0% (95% CI = 9.9-10.2); sibling fixed-effects: 8.5% (95% CI = 8.4-8.7); twin fixed-effects: 6.8% (95% CI = 5.3-8.3); MR: 10.1% (95% CI = 9.5-10.7)] (*SI Appendix,* Table S9, Panel C). The IRR indicates that despite negative early returns at younger ages, education pays off across the life cycle.

### Effect Heterogeneity.

To probe effect heterogeneity, we executed OLS and MR models in the full genotyped MoBa sample separately by sex and by parental earnings quartiles (*SI Appendix*, section 3.4.1). With respect to sex, both OLS and MR consistently indicated considerably higher returns to schooling on log earnings and absolute earnings for women than for men (*SI Appendix,* Table S8).

Findings were mixed with respect to effect heterogeneity by family origin. OLS, but not MR, models found larger returns to schooling on log earnings among individuals from low-income families. By contrast, MR, but not OLS, models found larger returns on absolute earnings among individuals from high-income families (*SI Appendix,* Table S8).

### Assessments of MR Assumptions and Sensitivity Analysis.

MR requires assumptions A1-A4 (stated above), which merit thorough assessment (*SI Appendix*, section 3.3).

#### Relevance (A1).

Assumption A1 requires that the PIV^EA^ strongly predicts years of schooling. Substantively, a one-SD increase in PIV^EA^ was associated with over a quarter of a year (0.26 y, 95% CI = 0.25-0.28) of additional schooling, net of covariates (*SI Appendix,* Fig. S2). We formally test A1 by examining the partial *F*-statistic on PIV^EA^ from the first-stage regression of years of schooling on PIV^EA^ and all control variables. The *F*-statistic exceeds 50 in all MR, sibling-MR, and Norway-only MR models (*SI Appendix,* Table S2), indicating that a) the MR point-estimates do not suffer weak-instrument bias (37), and b) the AR tests (52) for assessing the statistical significance of the point estimates are correct (37).

#### Independence (A2).

Assumption A2 requires the absence of unmeasured instrument-outcome (PIV^EA^-earnings) confounding (42, 53–56). We relaxed and examined A2 in multiple ways. Our sibling-MR and Norway-only MR analyses substantially relax A2 compared to the conventional MR model by adjusting for shared, between-family unobserved PIV^EA^-earnings confounding (including population stratification, assortative mating, and dynastic effects) in complementary ways. Sibling-MR adjusts for shared confounding by adding sibling fixed-effects in the estimation stage (22, 25, 28, 29); and the Norway-only MR adjusts for shared confounding in the construction of the PIV^EA^ from a within-sibling FGWAS (42). As shown in Fig. 2, like MR, the point estimates from sibling-MR and Norway-only MR exceed the OLS estimates. This suggests that possible violations of A2 are unlikely to account for the difference between our main MR and OLS results.

Since even our sibling-MR and FGWAS-based Norway-only MR estimates cannot fully rule out residual nonshared, individual-level unobserved confounding (56), we also executed between- and within-family covariate balance checks as falsification tests for A2 (38): If the PIV^EA^ is as good as random, then the PIV^EA^ should not strongly associate with covariates (38). The results reported in *SI Appendix*, Fig. S3 fail to find strong associations between PIV^EA^ and covariates in the samples for our MR, sibling-MR, and Norway-only MR models. Finally, to adjust for any lingering A2 violations related to observed covariates, all MR models adjust for observed covariates.

#### Exclusion (A3).

Assumption (A3) requires the absence of pleiotropic direct effects of PIV^EA^ on earnings. Owed to the central importance of this assumption, we performed an extensive set of analyses to detect and assess the presence and potential impact of pleiotropy, including multiple summary-level MR models that a) can test for and b) are robust to various forms of pleiotropy (22, 23), and a formal sensitivity analysis (41). Summary-level MR models aggregate independent SNP-specific MR estimates for the returns to schooling into an overall estimate (23). The baseline against which summary-level MR models should be compared to assess the impact of pleiotropy is the inverse-variance weighted (IVW) MR estimate (22, 23, 57) of 0.049 log points (95% CI = 0.045-0.053, *SI Appendix,* Table S4), i.e., 5.0%. Note that the IVW-MR estimate may differ from the MR estimate in Fig. 2 for multiple reasons and is not itself robust to pleiotropy (23, 58). Cochran’s *Q-*statistic for the IVW-MR estimate indicated heterogeneity across SNP-specific estimates (*SI Appendix,* Fig. S6), which could indicate pleiotropy in some SNPs (but not necessarily bias in the IVW-MR estimate) under the additional assumption of constant returns to schooling across the population (22, 23, 26).

MR-Egger models test and adjust for pleiotropy under the assumption that the instrument strength across SNPs is independent of their pleiotropic direct effects (59). The MR-Egger intercept test for directional pleiotropy was not statistically significant (*α* = −8.9 × 10^−4^, 95% CI = −1.8 × 10^−3^–1.0 × 10^−6^). MR-Egger produced a returns to schooling estimate of 0.065 log points (95% CI = 0.048-0.081, *SI Appendix,* Fig. S4 and Table S4), i.e., 6.7%, and overlapped with the baseline IVW-MR estimate. Leave-one-out MR-Egger analysis detected no outliers among SNP-specific estimates (*SI Appendix,* Fig. S5). Therefore, MR-Egger did not detect any evidence for bias due to pleiotropy.

The MR-Median estimator (60) permits that up to half of the information used in the model comes from pleiotropic SNPs. MR-Mode estimators (61) are robust to pleiotropic outliers under the assumption that the most common SNP-specific estimates are not pleiotropic. The MR-Corge estimator (39) is robust to pleiotropic outliers under the assumption that the SNP-specific estimates from the SNPs most strongly associated with EA are not pleiotropic. The estimated returns to schooling from MR-Median, MR-Mode (unweighted and simple), and MR-Corge were all positive, statistically significant, and overlapped with the baseline IVW-MR estimate (*SI Appendix,* Figs. S4 and S7 and Table S4). Therefore, none of our pleiotropy-robust estimators provided empirical evidence of pleiotropy bias in our main MR estimates.

#### Sensitivity analysis.

Since the summary-level MR estimators relax but do not fully obviate the A3 exclusion assumption, we also conducted a formal union of CI (UCI) sensitivity analysis (41) that investigated the robustness of our main MR estimates across a range of hypothetical exclusion violations. Centrally, we found that the direct (pleiotropic) effect of the PIV^EA^ on earnings would have to exceed NOK 12,650 to reduce the main MR estimate (from Fig. 2, Panel *B*, line 5) to statistical insignificance (*SI Appendix,* Fig. S9). Since the overall reduced-form association between PIV^EA^ and earnings, net of covariates, was NOK 14,133 (95% CI = 12,227-16,039), this shows that the MR estimate for the returns to schooling would remain positive and statistically significant even if nearly all (i.e., 12,650/14,133 = 89.5%) of the association between the PIV^EA^ and earnings were owed to an exclusion violation. This amount of pleiotropy appears unlikely, since the PIV^EA^ was specifically optimized to predict EA.

#### Monotonicity (A4).

Assumption A4 requires that the PIV^EA^ associates with schooling in the same direction for all sample members. A4 implies the weaker statement that all covariate weights in the MR-OLS decomposition (36) be positive. *SI Appendix,* Table S7 shows that this is the case, thus failing to falsify the A4 assumption.

### MR-OLS Decomposition.

We execute an MR-OLS decomposition (36) to investigate possible explanations for the difference between our MR and OLS estimates for the returns to schooling (*Materials and Methods* and *SI Appendix*, section 3.3.4).

Most of the MR-OLS gap (83.3%, *SI Appendix,* Table S6) is due to a combination of unobserved confounding in OLS and the difference in the average returns to schooling among compliers estimated by MR and the average returns in the general population estimated by OLS. The relative contributions of confounding vs. differential averaging cannot be distinguished (36).

Some of the MR-OLS gap (12.5%, *SI Appendix,* Table S6) also results from placing different weights on different schooling levels. MR placed slightly higher weights on college education than OLS (*SI Appendix,* Fig. S10). We note that both OLS and MR draw information from all schooling transitions (especially between 10 and 18 y of schooling), which contrasts with school-reform based IV estimates that refer to one particular additional year of schooling mandated by law (7).

Difference in the weighting of covariate-specific average effects across MR and OLS explains very little (4.2%, *SI Appendix,* Tables S6 and S7) of the gap.

### Labor Market Experience.

While labor market experience is commonly included as a covariate in standard Mincer-type equations for the returns to schooling, we excluded it because posttreatment covariates can pose identification challenges (62). Supplementary analyses that adjust for years of labor market experience found similar yet slightly higher returns across all identification strategies, in line with prior work (7) (*SI Appendix,* Fig. S11).

## Discussion

### Summary.

We estimated the economic returns to schooling from population-wide registry and genetic data across multiple, increasingly rigorous statistical approaches. Estimates were smallest in fixed-effects models, larger in OLS, and largest in MR. Our key results were remarkably consistent across designs in three respects. First, prime-age returns to schooling (ranging from 3.2 to 9.7% for an additional year) were positive, statistically significant, and large (Fig. 2 and *SI Appendix,* Table S2) (51). Second, life-cycle analyses showed that returns to schooling varied by age. Returns were negative at young ages due to forgone labor market experience and earnings, became positive around age 27, and kept growing until the end of workers’ careers (Fig. 3 and *SI Appendix,* Table S9). Third, the lifetime returns to schooling as measured by the IRR (ranging from 6.8 to 10.1%, *SI Appendix,* Table S9) exceeded opportunity costs as proxied by the market interest rate (2.3%). Regardless of the estimation strategy, the evidence showed that education pays off.

### Genetically Informed MR Estimates.

A key contribution of our research is the use of genetically informed designs that exploit the genetic lottery to generate quasi-experimental MR estimates for the returns to schooling. We offer the MR results as our preferred specification, for two reasons: causal credibility and generality.

First, MR estimates adjust for all sources of unobserved confounding under IV assumptions (22, 23, 26). By contrast, conventional OLS adjusts only for observed confounding, and sibling and twin fixed-effects models only adjust for observed and shared sibling- and twin-level unobserved confounding, respectively. Neither OLS nor fixed-effects models can adjust for individual-level unobserved confounding. While researchers can never verify that all necessary assumptions hold (26, 27), our MR results held up to a comprehensive battery of tests, falsification tests, robustness checks, and a formal sensitivity analysis (*SI Appendix*, section 3.3), suggesting that MR is valid for identifying the causal returns to schooling.

Second, our MR results are more general than other causally credible estimates of the returns to schooling that exploit school reforms. Whereas school-reform based estimates only identify the effect of changing the amount of mandatory schooling (5, 7), our MR-OLS decomposition (36) demonstrates that our MR estimates capture the effect of any amount of additional schooling observed beyond mandatory schooling (*SI Appendix*, section 3.3.4).

### Comparison of Estimates.

Our finding that MR estimates are higher than our fixed-effects and OLS estimates (Fig. 2) replicates the well-known pattern that IV (including MR) estimates for the returns to schooling exceed more conventional estimates (28, 29, 63). We considered six possible explanations.

First, there may be negative unobserved confounding at the individual level, which would negatively bias fixed-effects and OLS but would not bias MR. Negative unobserved individual-level confounding would occur if enough individuals with unobserved high-earnings potential leave school early (62). This is plausible in Norway, where a compressed wage structure and rich natural resource industries offer well-paying career paths at low levels of schooling to individuals with strong nonacademic skills or entrepreneurial talent (64, 65).

Second, the target populations may differ across estimates. Whereas OLS targets the entire population and fixed-effects target families in which siblings or twins receive different amounts of education, MR targets compliers, i.e., individuals who are induced by the PIV^EA^ to receive more education (22, 26). MR estimates would be larger than OLS and fixed-effects estimates if compliers enjoy systematically higher returns to education. Our MR-OLS decomposition provides some evidence for this possibility, because our MR estimates place greater weight on individuals with tertiary education and especially graduate education (*SI Appendix,* Fig. S10) and on individuals who have a lower socioeconomic background (*SI Appendix,* Table S7), both of which are related to higher returns to schooling (18, 66).

Third, MR may be upwardly biased if the SNPs in the PIV^EA^ exert a positive pleiotropic effect on earnings that does not operate via increased schooling (26). Although we find no empirical evidence for bias due to pleiotropy, and we also find that pleiotropy would have to be implausibly strong to reduce our MR estimates to statistical insignificance (*Results and Limitations*), we cannot rule out the presence of any pleiotropic upward bias in our MR estimates.

Fourth, all estimates may be biased due to minor differential measurement error. While educational attainment is obtained from rigorously audited administrative registries, years of schooling may be understated for individuals who do not complete their degree program. It is difficult to predict the direction of the bias from differential measurement error (67).

We cautiously discount two additional explanations. Fifth, sample differences are unlikely to explain differences in estimates because OLS estimates for the association between schooling and prime-age log earnings agree closely across samples (within 0.01 log points, *SI Appendix,* Table S3). Sixth, positive confounding between the PIV^EA^ and earnings is unlikely to explain higher MR estimates because our sibling fixed-effects MR and FGWAS-based Norway-only MR estimates adjust for family-level unobserved confounding yet still exceed OLS and fixed-effects estimates (Fig. 2).

### Limitations of MR.

MR requires the assumptions of A1 relevance, A2 independence, A3 exclusion, and A4 monotonicity. Extensive probes of these assumptions detected no evidence to challenge their validity (see Assessment of MR Assumptions and *SI Appendix*, section 3.3). However, only A1 is fully testable, whereas A2, A3, and A4 are not.

Large first-stage *F*-statistics (*SI Appendix,* Table S2) indicate that A1 relevance is valid for all models presented in Fig. 2.

The A2 independence assumption could be invalid to the extent that there remains nonshared individual-level unobserved confounding of the PIV^EA^-earnings association after adjusting for family-based confounding in sibling-MR. This would be of concern even in our FGWAS-based Norway-only MR estimate if individual-level genetic confounding in the PIV^EA^-EA association also affects earnings. Recent theoretical models, however, suggest that such confounding in FGWAS-generated PIVs is typically minimal (43, 56). A2 might also be violated if selection into the MoBa genotyped sample is associated with PIV^EA^ and unadjusted predictors of earnings. However, since selection into MoBa is determined by parents, our sibling-MR models effectively obviate this concern.

The A3 exclusion assumption could be invalid to the extent that there remain exclusion violations, e.g., due to pleiotropic direct effects of the PIV^EA^ on earnings, e.g., via fields of study (68, 69), health conditions (70), or personality traits (69), that are not detected or adjusted by our pleiotropy-robust MR-Egger, MR-Mode, MR-Median, and MR-Corge models (*SI Appendix*, section 3.3.2). We cannot fully rule out such violations empirically or theoretically because the pleiotropy robust models rely on auxiliary identifying assumptions of their own (59–61), and because knowledge of the biological and social mechanisms linking the SNPs to EA is incomplete and evolving (24, 71). While the UCI sensitivity analysis (41) demonstrates that the MR estimate would remain positive and statistically significant even if almost the entire PIV^EA^-earnings association were due to a pleiotropic direct effect of the PIV^EA^ on earnings (*SI Appendix*, section 3.3.3), smaller amounts of pleiotropy could still bias our MR estimates, albeit without overturning the qualitative conclusion of positive returns to education.

The A4 monotonicity assumption could be invalid to the extent that monotonicity is defined with respect to the direction of the individual-level causal effects of the PIV on EA, but our empirical analysis (*SI Appendix*, section 3.3.4) only assesses the average direction of the effect within strata defined by key covariates.

One might additionally voice concerns with our main MR and sibling-MR models because their PIV^EA^, as in related works (28, 29, 72), is constructed from the SNPs and their effect sizes in the international EA4 GWAS (24), which may not be portable to Norway (53). Our Norway-only MR models address portability by constructing a new PIV^EA^ from the SNPs in EA4 (24) and SNP-effect sizes estimated in an exclusively Norwegian discovery sample (Fig. 2 and *SI Appendix*, section 1.3). If EA4 contains SNPs that do not act on EA in Norway, using Norwegian effect sizes would effectively drop these irrelevant SNPs. If EA4 misses some SNPs that do act on EA in Norway, this would only reduce the statistical power of our Norway-only MR estimates and diminish their precision.

Finally, the generalizability of our MR estimates may be limited in three respects. First, our MR models analyze the MoBa cohort study, whose participants are healthier and more socioeconomically advantaged than the average Norwegian (73). However, prior work supports the generalizability of associations from MoBa to the general population (34, 74, 75). Second, our study was conducted in the Norwegian universal welfare state with nearly free education and other policies aimed at reducing barriers to education. Therefore, our findings may not generalize to other institutional and national contexts. Third, gains to schooling may vary across individuals in ways not captured in our heterogeneity analysis (*SI Appendix*, Table S8), e.g., by fields of study (68).

## Conclusions

Rising educational attainment and technological changes have intensified debates about the economic value of education (76, 77). This study advances the returns to schooling literature by demonstrating how the careful integration of genetic data can help investigate hard-to-answer causal questions in the social sciences. Our MR approach exemplifies insights gained from combining genetic data with quasi-experimental designs. By exploiting the genetic lottery at birth as an instrument, we estimate that one additional year of schooling results in 8% higher annual earnings in our main MR model. While conventional approaches yield lower estimates, the IRRs for all approaches exceed opportunity costs of education as proxied by the market interest rate. These findings reinforce confidence in the economic value of education, with important implications for individual educational decisions (1) and policy development (2).

## Materials and Methods

### Data.

We used microdata and genotyped data from MoBa, twin data from the Norwegian Twin Registry (NTR), and multiple nationwide registers maintained by Statistics Norway (SSB) (*SI Appendix*, section 1). We used genetic data for parents in MoBa from MoBaPsychGen pipeline v.1 (78). Genomic data are obtained from blood samples from parents during pregnancy (79). MoBa is a population-based pregnancy cohort study conducted by the Norwegian Institute of Public Health. Participants were recruited from all over Norway from 1999 to 2008. MoBa is based on informed consent from all participants in accordance with Norwegian regulations on population-based health surveys (80). The women consented to participation in 41% of the pregnancies. In 87.3% of participating pregnancies, fathers were also invited to participate, with 82.9% providing consent (74). The cohort includes approximately 114,500 children, 95,200 mothers and 75,200 fathers. MoBa is regulated by the Norwegian Health Registry Act. The current study was approved by The Regional Committees for Medical and Health Research Ethics (2017/2205). Demographic data and family ties are from the Norwegian central population register. Educational attainment for individuals and their parents is retrieved from the National Education Database (NUDB). Earnings data are from tax records collected by the Norwegian Tax Administration.

### Samples and Measurement.

We analyze six main samples (*SI Appendix,* Fig. S1): The full population sample (N = 1,255,604), full sibling sample (N = 966,976), twin sample (N = 5,849), MoBa genotyped sample (N = 109,800), MoBa genotyped siblings sample (N = 18,666), and the MoBa genotyped nonsibling sample (N = 89,179). Labor market earnings was measured as the mean of the top three earnings years during ages 34 to 40, providing a measure strongly related to lifetime earnings (45, 81) (*SI Appendix*, section 1.3). Years of schooling was measured as the highest educational attainment when aged 33, ranging from 6 to 23 y (*SI Appendix*, section 1.3). Across samples, we used birth cohorts 1959-1982 as they allowed measurement of key variables at important stages of the life-cycle [i.e., earnings over multiple years (45), education when most have completed their schooling, family characteristics during childhood], and cover most genotyped MoBa participants (88%). We included several covariates for demographic and family characteristics (*SI Appendix,* Table S1).

The samples are subject to the following restrictions. First, the genotyped MoBa sample consists of parents in MoBa with quality-controlled genotyped data for PIV^EA^. Second, since genotyped data are restricted to European ancestry and we use long earnings panels, the convention of restricting to Norwegians is followed (7, 81, 82). Third, individuals must have valid data on earnings, education, and their mother and father must be registered for family-based data.

We constructed a PIV^EA^ using weights and direction of effects of variants identified in the EA4 GWAS (24), excluding 23andMe and MoBa participants. We excluded SNPs not available in MoBa and the 1000 Genome Project reference set. Using Plink 1.9, we then identified variants independently associated with EA, with a strict clumping threshold of *r*^2^ < 0.001, LD = 10,000 kb, at *P* < 5.0 × 10^–8^ following recommendations for MR-analyses (40). This left 335 SNPs associated with EA4 that we used to construct the allele scores for individual participants (*SI Appendix*, section 1.3). As a sensitivity analysis addressing potential concerns of portability across countries, we conducted an FGWAS in MoBa following the methodology of Howe et al. (44) and Okbay et al. (24) for phenotype definition. We used our MoBa siblings sample as the discovery sample [N = 21,182, participants with a kinship coefficient between 1/2^(3/2)^ and 1/2^(5/2)^ and a probability of zero IBS sharing > 0.0012 (83)]. Effect sizes from the FGWAS were used to construct a Norwegian-specific PIV^EA^ based on the same 335 SNPs as EA4 (24). The Norwegian-specific PIV^EA^ was applied in our unrelated MoBa sample (N = 89,179, kinship coefficient < 1/2^(9/2)^) for estimation to ensure separation between discovery and estimation.

### Statistical Analyses.

We estimated returns to schooling using several methods. First, we used OLS as an observational benchmark. Second, we used sibling and twin fixed-effects. Third, we estimated MR using 2SLS. Fourth, we estimated sibling-MR using fixed-effects IV models. Fifth, we estimated Norway-only MR using 2SLS. Details on samples are in *SI Appendix*, section 1 and model specifications in *SI Appendix*, section 2. We tested the statistical significance of MR point estimates using AR tests (37) and computed MR CI by inverting these tests.

MR assumptions A1-A4 were examined with a battery of tests, falsification tests, sensitivity analyses, and robustness checks, described above and in *SI Appendix*, section 3.3 and thus only briefly summarized in the following. The relevance assumption (A1) is examined with weak-instrument diagnostics (37). The independence assumption (A2) is addressed by 1) adjustment for key covariates (e.g., parental education and earnings) (26), 2) estimating sibling-MR to adjust for family-level unobserved confounding (22, 25, 28, 29), 3) estimating Norway-only MR using a PIV^EA^ constructed from a FGWAS within siblings in Norwegian data (42–44, 53), and 4) conducting covariate-balance tests (38, 84). The exclusion assumption (A3) is tested, assessed, and addressed by 1) using strongly associated genetic variants to minimize potential pleiotropic variants (39, 40), 2) the MR-Egger intercept test (23), 3) executing pleiotropy-robust summary-level MR analyses (MR-Median, MR-Mode, and MR-Corge) (22, 23, 40), 4) a formal sensitivity analysis of robustness to a range of pleiotropic direct effects (41). The monotonicity assumption (A4) is examined through covariate-specific weights (36).

Under MR assumptions A1-A4, and with a continuous instrument and treatment, our MR design identifies a generalized LATE (26). Specifically, our MR estimate identifies the weighted average effect of one additional year of schooling on earnings for individuals who complete more schooling only due to having a higher genetic predisposition for educational attainment. This subgroup, representing individuals on the margin of additional schooling, is the continuous analogue to compliers in the binary LATE framework (26).

To estimate returns to schooling across the life-cycle and the lifetime returns to schooling, we implemented the methodology of Bhuller, Mogstad, and Salvanes (7) (*SI Appendix*, section 3.5). Effect heterogeneity was examined with stratification by sex and parents’ earnings. The gap between estimated returns to schooling between MR and OLS was investigated with an MR-OLS decomposition method (36).

## Supplementary Material

Appendix 01 (PDF)

## Data Availability

Code is available on GitHub (https://github.com/tarjeiw/mr-iv-returns). We follow the STROBE-MR reporting guidelines (85). Data availability for research purposes is subject to strict Norwegian privacy regulations. Details on access to microdata can be obtained from mikrodata@ssb.no and datatilgang@fhi.no.
