# Supplementary material for: Estimating returns to education using the genetic lottery
Source: Proc Natl Acad Sci U S A. 2026 Apr 8;123(15):e2537049123. doi: 10.1073/pnas.2537049123 (PMC13079366; doi:10.1073/pnas.2537049123)
Supplement: Supplementary file 1 — Appendix 01 (PDF) [file pnas.2537049123.sapp.pdf]

PNAS

Supplementary information for

Estimating returns to education using the genetic lottery

Tarjei Widding-Havneraas, Perline A. Demange, Henrik Daae Zachrisson, Nicolai T. Borgen, Eivind Ystrom, Felix Elwert

Corresponding author: Tarjei Widding-Havneraas (tarjei.widding-havneras@create.uio.no)

This PDF file includes: Supporting text, Figures S1 to S12, Tables S1 to S9

Table of Contents

1. Sample and data 2
 1.1 Sample construction 2
 1.2 The Norwegian Mother, Father and Child Cohort Study (MoBa) 3
 1.3 Measurement 3
2. Model specifications 6
3. Results 8
 3.1 Descriptive statistics 8
 3.2 Returns to schooling estimates 9
 3.3 Assessing MR assumptions 11
  3.3.1 Relevance (A1) and independence (A2) 11
  3.3.2 Exclusion (A3): Summary-level MR to test and adjust for pleiotropy 14
  3.3.3 Exclusion (A3): Sensitivity analysis 22
  3.3.4 Monotonicity (A4) and MR-OLS decomposition 24
 3.4 Additional analyses 29
  3.4.1 Effect heterogeneity 29
  3.4.2 Labor market experience 30
 3.5 Life-cycle earnings and the internal rate of return 32
  3.5.1 Age-specific returns to schooling and the internal rate of return (IRR) 32
  3.5.2 Data for lifetime returns to schooling analyses 34
  3.5.3 Lifetime returns to schooling and IRR 35
  3.5.4 Sensitivity analysis: Balanced panel 37
4. Statistical software 38
5. References 39
6. STROBE-MR checklist 42

1. Sample and data

1.1 Sample construction

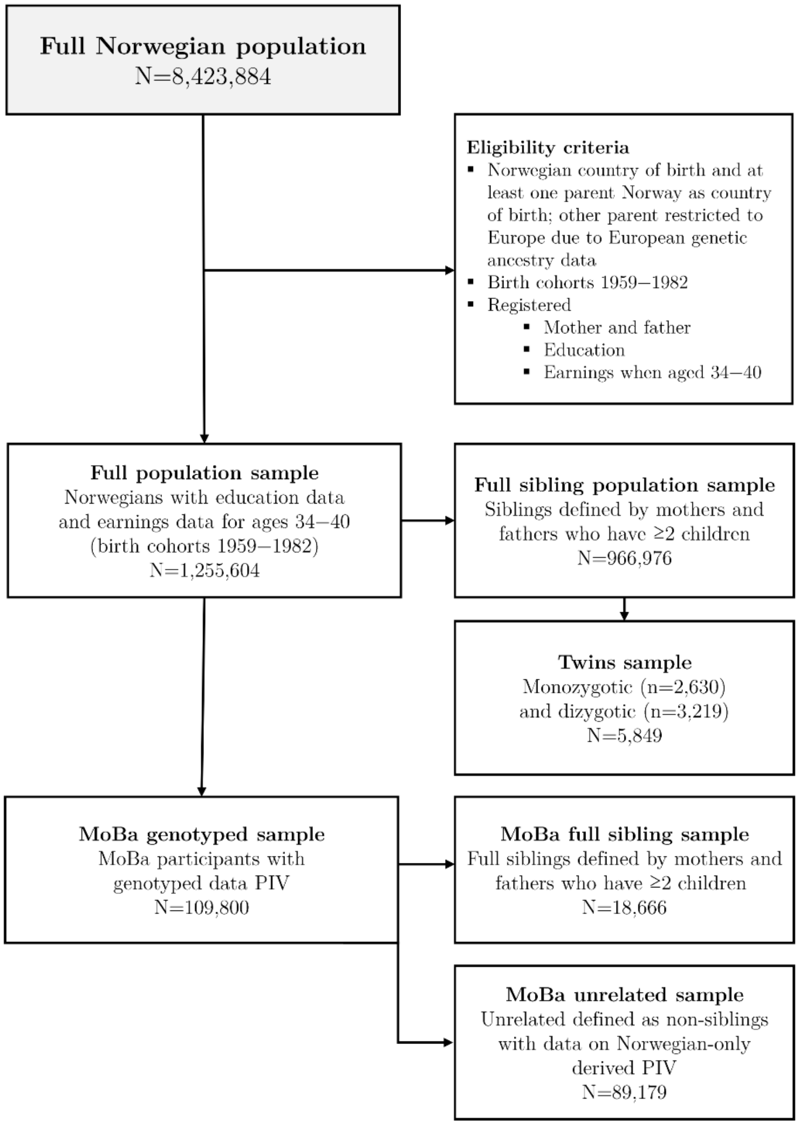

Figure S1. Flowchart for sample construction.

1.2 The Norwegian Mother, Father and Child Cohort Study (MoBa)

MoBa is a population-based pregnancy cohort study conducted by the Norwegian Institute of Public Health. Participants were recruited from all over Norway from 1999-2008. MoBa is based on informed consent from all participants in accordance with Norwegian regulations on population-based health surveys (1). The women consented to participation in 41% of the pregnancies. In 87.3% of participating pregnancies, fathers were also invited to participate, with 82.9% providing consent (2, 3). The cohort includes approximately 114,500 children, 95,200 mothers and 75,200 fathers. MoBa is regulated by the Norwegian Health Registry Act. The current study was approved by The Regional Committees for Medical and Health Research Ethics (2017/2205). The current study is based on version 12 of the quality-assured data files released for research in 2019.

1.3 Measurement

Earnings

We used individual-level annual data available for 1967-2022 allowing a consistent measure of earnings for individuals and their parents (4). We measured earnings as pre-tax pension-point generating earnings, which equal the sum of income from wages, self-employment, and fixed-term work-related transfers (e.g., unemployment benefits, short-term sickness benefits, and parental leave) in the seven-year period when individuals are aged 34-40. To reduce the potential influence of early labor market exit, years with low or no labor market earnings and participation, we used a "best year rule", averaging the top three earnings years for ages 34-40 (4, 5). These ages were chosen as they are strongly related to lifetime earnings ($r \approx .7$) (4), while allowing us to use most birth cohorts with genotyped data in MoBa. We thus estimate the marginal effect of schooling on earnings at ages strongly correlated with lifetime earnings (4).

Earnings are inflation-adjusted to NOK 2022-levels using the annual wage index (full time equivalent annual wage) (6, 7). Due to earnings registration some high earnings may be registered with low or no earnings, and as there is no strict top-coding we trim the lowest and highest two percent. We log our average earnings measure consistent with the standard Mincer equation, thereby obtaining a more normally distributed outcomes variable and the convenient interpretation of percentage-wise changes in earnings with additional schooling (8, 9). Our lifetime analyses for age-specific education premiums follow the approach by Bhuller, Mogstad and Salvanes (6) (details in Section S3.5).

Education

Educational attainment is measured as years of completed schooling based on the NUS2000 classification and with the following categories (10, 11): primary school (PS; 6 years), lower secondary school (LSS; 9 years), some high school HS (10 years), complete HS (12 years), extra HS (13 years), bachelor's degree (BA; 16 years), master's degree (MA; 18 years), and PhD (23 years). We used years of completed schooling by age 33. Educational attainment is registered and reported by institutions to Statistics Norway, ensuring negligible measurement error (6). For our within-sibling family-based GWAS (FGWAS) in MoBa, we use the same definition of years of education as Okbay et al. (12), which, following common practice, converts the Norwegian NUS2000 classification into years of education in line with the US schooling system.

Polygenic instrumental variable

Our individual-level MR analyses use independent genetic variants highly associated with educational attainment (EA) combined in a polygenic index for EA as an IV, which we term polygenic instrumental variable (PIV^{EA}). The PIV^{EA} is the numeric summary of many single-nucleotide polymorphisms (SNPs) weighted by their so-called effect sizes (coefficients) on EA from a genome-wide association study (GWAS) (13). Following Mills, Barban and Tropf (13), let the individual-specific PIV^{EA} be the weighted sum of the alleles,

$$PIV_i^{EA} = \sum_{j=1}^{K} a_{ij}\, w_j \tag{1}$$

where $a_{ij} \in (0,1,2)$ are the alleles for individual i and SNP $j = 1, \ldots, K$, and weights w_j are the GWAS coefficients for each allele.

We constructed PIV^{EA} using weights and direction of effects of variants identified in the EA4 GWAS (12), excluding 23andMe and MoBa participants. We excluded SNPs not available in MoBa. Using Plink 1.9, we then identified variants independently associated with EA, with a clumping threshold of r^2<0.001, *LD*=10,000 *kb* at p<5.0×10–8. This left 335 SNPs associated with EA, which we used to construct the PIV^{EA} for each individual participant, with Plink 1.9. Strict clumping threshold of r^2<0.001 follows the current two-sample MR R package recommendation (14). This strict clumping and thresholding strategy to identify genetic instruments was done to limit potential violation of the exclusion restriction (assumption A3). While such a strict threshold reduces the number of SNPs included in the PIV^{EA}, which might lower the predictive power of the PIV^{EA}, it also eliminates the weakest instruments among the

alleles, and thus likely reduces the risk of weak instrument bias and the risk of horizontal pleiotropy (13, 15). We use the same 335 SNPs in our summary-level MR analyses.

Norway-specific polygenic instrumental variable

To address potential concerns about the portability of genetic effect sizes from the EA4 international discovery sample (12) to the Norwegian population, we constructed a Norway-specific PIV^{EA} using weights derived from a within-sibling FGWAS in MoBa, following the methodology of Howe*, et al.* (16) and Okbay*, et al.* (12). We used our MoBa siblings sample as the discovery sample (N=21,182, participants with a kinship coefficient of $1/2^{(3/2)}$ and $1/2^{(5/2)}$ and a probability of zero IBS sharing > 0.0012 (17)). We then used effect sizes from this FGWAS to construct a Norwegian-specific PIV^{EA} based on the same 335 SNPs from EA4 (12). We used this Norwegian-specific PIV^{EA} to estimate an MR model in an unrelated MoBa sample (N=89,179, kinship coefficient < $1/2^{(9/2)}$) to ensure separation between discovery and estimation. We term the analysis that uses this Norwegian-specific PIV^{EA} the Norway-only MR model.

Covariates

Our covariates include sex, birth year, birth month, and birth order, and several measures for family characteristics. Parent's earnings were measured as the mean of both parents' average earnings based on each parent's top three earnings years when the child was aged 8 to 14 years, and divided into vigintiles (5% groups), included in linear and quadratic form (4, 5). Parents' education was measured as the average years of schooling for both parents when the individual was aged 11, included in linear and quadratic form. Father's or mother's earnings vigintile and education level was used if information was not available for both parents. Parents age at birth is the mean of both parents' age when the individual was born (categories: ≤ 19; 20-24; 25-29; 30-34; 35-39; ≥ 40). Number of children in the family is the total number of children in the family.

2. Model specifications

We modelled earnings as a linear function of years of schooling as a Mincer equation (or human capital earnings function) (8), separately for log earnings and monetary units (NOK).

OLS with covariate-adjustment. Focusing on log earnings (replaced by unlogged $e$arnings in levels equations) for our full-population sample, we have

$$log(Y_i) = \beta_0 + \beta_1 S_i + X_i' \beta_2 + \varepsilon_i \quad (2)$$

where earnings are denoted Y, years of schooling S, and covariates X, and other unobserved factors reflected in the error term ε. Here, β_0 is the intercept, β_1 is the returns to schooling, and β_2 is a vector of coefficients on the covariates. Covariates include parents' earnings (and its squared term), parents' years of final schooling (and its squared term), and as set of indicators: sex, birth year, birth month, parents' age at birth and number of children in the family.

Sibling fixed-effects model. In our sibling fixed-effects model, we restrict the sample to groups of ≥ 2 full siblings and enter family-level fixed-effects. For each full sibling in family j, we isolate within-family variation in earnings and schooling, to remove shared family-level unmeasured confounding (e.g., parental genetics, shared family environment, school, and neighborhood). Since sibling covariates can differ for different-age siblings, the same covariates as our OLS model are included except for number of children in the family (constant). We estimate

$$log(Y_{ij}) = \beta_0 + \beta_1 S_{ij} + X_i' \beta_2 + \zeta_j + \varepsilon_{ij} \quad (3)$$

where β_1 is interpreted as the average within-family returns to schooling and ζ_j are family fixed-effects.

DZ and MZ fixed-effects twin models. The twin analysis follows Eq. 3, where the sample is restricted to DZ and MZ twins, respectively. Since twins are born at the same time, only sex is included among covariates in the DZ model.

Mendelian randomization. We estimate all MR models with two-stage least squares (2SLS), where equation (4) is the first stage and equation (5) is the second stage,

$$S_i = \beta_0 + \beta_1 Z_i + X_i' \beta_2 + \varepsilon_i \quad (4)$$

$$log(Y_i) = \delta_0 + \delta_1 \hat{S}_i + X_i' \delta_2 + \nu_i \quad (5)$$

where Z is the PIV^{EA}, entered as a continuous variable that captures the individual's genetic propensity for educational attainment. Under the MR assumptions A1-A4 stated in the main manuscript, heterogenous effects, and without covariates, the 2SLS estimate of δ_1 identifies the average effect of one additional year of schooling among persons who complete one additional year of schooling due to having a higher genetic propensity for schooling (i.e., the continuous extension to the well-known local average treatment effect (LATE)). Covariates include parents' earnings (and its squared term), parents' years of final schooling (and its squared term), and as set of indicators for sex, birth year, birth month, parents' age at birth and number of children in the family. With covariates, MR recovers a weighted average effect across covariate-specific subgroups (18).

Sibling Mendelian randomization. In our sibling-MR model, we restrict the sample to groups of ≥ 2 full siblings and enter family-level fixed-effects. The model for sibling MR analyses with family-level fixed-effects ζ_j is

$$S_{ij} = \beta_0 + \beta_1 Z_{ij} + X_{ij}' \beta_2 + \zeta_j + \varepsilon_{ij} \quad (6)$$

$$log(Y_{ij}) = \delta_0 + \delta_1 \hat{S}_{ij} + X_{ij}' \delta_2 + \zeta_j + \nu_{ij} \quad (7)$$

Since sibling covariates can differ by birth cohort for different ages, the same covariates as our MR model are included except for number of children in the family (constant). For all models, we report models with and without covariate adjustment. Adjusted analyses were conducted on complete cases with nearly no missing data (<1%). Standard errors are clustered by family and birth cohort in family (sibling and twin) models, and by birth cohort otherwise. For MR, we report Anderson-Rubin (AR) tests and 95% confidence intervals (19).

3. Results

3.1 Descriptive statistics

	Full population sample		Full population sibling sample		Twins sample		MoBa genotyped sample		MoBa genotyped sibling sample		MoBa genotyped unrelated sample	
	(1)		(2)		(3)		(4)		(5)		(6)	
	Mean	SD	Mean	SD	Mean	SD	Mean	SD	Mean	SD	Mean	SD
Median earnings, aged 34-40‡	638,368	(321,852)	639,947	(320,233)	657,492	(305,385)	664,702	(318,388)	668,913	(320,475)	663,695	(317,002)
Years of schooling, age 33	13.02	(2.99)	13.03	(2.96)	13.88	(3.02)	14.40	(2.78)	14.72	(2.66)	14.33	(2.80)
Birth year	1970.02	(6.71)	1969.89	(6.25)	1971.11	(6.86)	1973.20	(4.75)	1973.36	(4.30)	1973.16	(4.83)
Birth month	6.37	(3.39)	6.36	(3.39)	6.40	(3.37)	6.36	(3.39)	6.31	(3.39)	6.37	(3.39)
Men (proportion)	0.51	-	0.51	-	0.42	-	0.40	-	0.39	-	0.40	-
Parents' total earnings ventile†	10.48	(5.82)	10.42	(5.77)	11.37	(5.88)	11.62	(5.67)	11.72	(5.63)	11.59	(5.68)
Parents' years of schooling	11.23	(2.17)	11.25	(2.15)	11.76	(2.46)	11.85	(2.35)	12.16	(2.40)	11.78	(2.31)
Parents' age at birth	28.45	(5.83)	28.15	(5.40)	29.63	(5.48)	27.93	(5.38)	27.89	(4.87)	27.93	(5.47)
Number of children in family	2.27	(1.01)	2.65	(0.83)	3.00	(0.91)	2.27	(0.92)	2.79	(0.85)	2.17	(0.90)
Birth order	1.64	(0.84)	1.83	(0.87)	-	-	1.71	(0.87)	1.96	(0.95)	1.67	(0.85)
N	1,255,604		966,976		5,849		109,800		18,666		89,179	

Table S1. Summary statistics for population samples and genotyped MoBa samples
‡ Earnings reported as median with interquartile range. †Parents' total earnings is the sum of both parents average earnings when the individual was aged 8 to 14 years old, split into ventiles (groups of 5%). Parents' years of schooling the mean of both parents final schooling measured when the individual was aged 11, while parents age at birth is the mean of both parents' age when the individual was born. Number of children in family is the total number of children in the family.

3.2 Returns to schooling estimates

	Log (Earnings)		Earnings (NOK)			
Panel A: Returns to schooling estimates	Coef.	95% CI	Coef.	95% CI	Controls	Sample size
OLS	0.060***	(0.059-0.061)	37,247***	(36,758-37,736)	×	1,255,604
	0.058***	(0.056-0.059)	34,618***	(34,087-35,148)	✓	1,255,604
Sibling FE	0.051***	(0.051-0.052)	30,848***	(30,535-31,161)	×	966,976
	0.051***	(0.051-0.052)	30,825***	(30,511-31,139)	✓	966,976
Twin FE (DZ)	0.042***	(0.032-0.052)	26,194***	(19,510-32,879)	✓	3,219
Twin FE (MZ)	0.032***	(0.018-0.045)	19,196***	(11,113-27,280)	✓	2,630
MR	0.075***	(0.069-0.081)	52,941***	(45,991-56,708)	×	109,800
	0.077***	(0.069-0.086)	53,622***	(47,701-59,544)	✓	109,800
Sibling-MR	0.061**	(0.016-0.107)	45,779**	(14,082-81,674)	×	18,666
	0.061**	(0.015-0.110)	45,560**	(12,546-80,891)	✓	18,666
Norway-only MR	0.087***	(0.068-0.103)	59,682***	(46,866-70,761)	×	89,179
	0.093***	(0.062-0.121)	62,050***	(40,489-82,098)	✓	89,179
Panel B: IV first stage *F*-statistic	Kleibergen-Paap rk Wald *F* Statistic					
MR	2,570.7				×	
	1,104.3				✓	
Sibling-MR	61.8				×	
	60.8				✓	
Norway-only MR	299.2				×	
	117.6				✓	

Table S2. Returns to schooling estimates. Standard errors (SE) are clustered by family and birth cohorts in family models, and birth cohorts otherwise. The *F*-statistic assesses the strength of the first-stage in MR models. Anderson-Rubin (AR) 95% confidence intervals reported for MR, sibling-MR, and Norway-only MR. Controls include birth cohort, sex, parents' earnings, parents' years of schooling, parents' age at birth, birth order, number of children in family. Family size is omitted in sibling FE; covariates except sex omitted in the DZ twin model; and no covariates in the MZ twin model due to within-family invariance. Models without controls only adjust for sex and birth cohorts. Models based on same sample with complete data on covariates for comparison. NOK=Norwegian kroner; OLS=Ordinary least squares; IV=Instrumental variable; MR=Mendelian randomization; DZ=dyzigotic; MZ=monozygotic; FE=Fixed-effects. NOK in 2022 values. *** $p < 0.001$, ** $p < 0.01$, * $p < 0.05$.

Table S2 shows returns to schooling estimates with and without controls for our main OLS, fixed-effects, and MR models. The AR test statistic (15, 19, 20) was statistically significant for both MR (unadjusted AR: χ^2=16.99; adjusted AR: χ^2=17.04), sibling-MR (unadjusted AR: χ^2=5.6; adjusted AR: χ^2=5.5), and Norway-only MR (unadjusted AR: χ^2=13.72; adjusted AR: χ^2=11.64) models.

To assess sample comparability, Table S3 reports OLS estimates for the returns to schooling across samples, controlling only for sex and birth cohort. Point estimates are very similar across samples (within 0.01 log points). This suggests that the differences in the OLS, fixed-effects, and MR estimates on these samples, reported in Figure 2 and Table S2, are not due to differences in population composition across samples.

	Log (Earnings)	
OLS, adjusting only for sex and birth cohort	Coef.	SE
Full population sample	0.060***	(0.001)
Sibling sample	0.058***	(0.001)
DZ twins sample	0.054***	(0.003)
MZ twins sample	0.049***	(0.003)
MoBa genotyped sample	0.056***	(0.001)
MoBa genotyped sibling sample	0.053***	(0.001)
MoBa genotyped unrelated sample (Norway-only)	0.056***	(0.001)

Table S3. OLS estimates of returns to schooling across samples. Estimated returns to schooling from OLS models adjusted only for sex (except MZ Twins) and birth cohorts. Robust standard errors (SE) in parentheses. *** $p < 0.001$, ** $p < 0.01$, * $p < 0.05$.

3.3 Assessing MR assumptions

This subsection (3.3) presents our assessment of the MR assumptions, A1-A4, using various tools: tests, falsification tests, robust estimators, formal sensitivity analyses, and an MR-OLS decomposition. We execute these tests for our main MR analyses (MR with and without sibling fixed-effects) and for our Norway-only MR analyses that construct the PIV^{EA} from a Norway-only family-based within-sibling GWAS (FGWAS).

3.3.1 Relevance (A1) and independence (A2)

The MR analyses reported in Figure 2 combine independent genetic variants highly associated with educational attainment (EA) into a polygenic index for EA, which we use as our polygenic instrumental variable (PIV^{EA}). The PIV^{EA} was based on the 335 independent genetic variants from Okbay et al.'s (12) EA4 GWAS that were most strongly associated with EA to minimize possibly pleiotropic variants (13, 21).

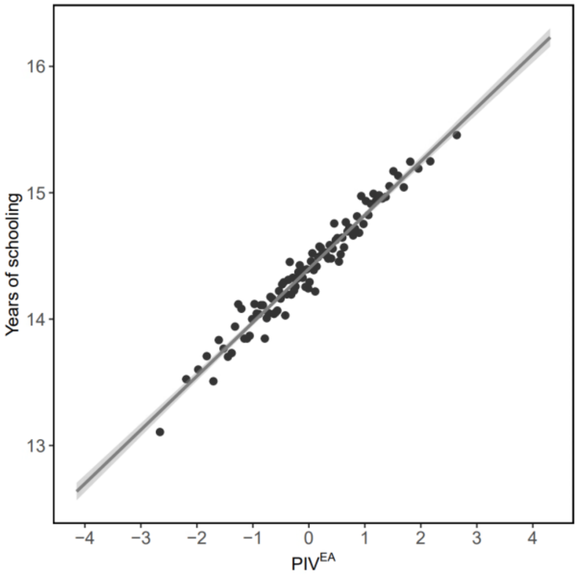

Figure S2. Association between polygenic instrumental variable for educational attainment and years of schooling. Binned scatterplot showing bin means (dots; number of bins=100) with fitted regression line and 95% confidence intervals (gray shading) using the full MoBa genotyped sample (*N*=109,800). PIV^{EA}=polygenic instrumental variable for educational attainment.

Relevance (A1): Assumption A1 ("relevance") requires that the PIV^{EA} is strongly associated with EA to avoid weak instrument bias and enable valid statistical inference. PIV^{EA} was strongly related to years of schooling (Figure S2). A one-standard deviation increase in PIV^{EA} was associated with over a quarter of a

year (coefficient=0.26, 95% CI=0.25-0.28) of additional schooling, net of covariates. The first-stage F-statistic in MR and sibling MR (Table S2) ranged from 61 (sibling-MR with covariates) to 2,571 (MR without covariates). These values comfortably exceed the latest thresholds (F=50) for valid statistical inference with AR tests (19). Therefore, the PIV^{EA} is not "weak" in any of our main models, supporting A1 ("relevance").

Independence (A2): Assumption A2 ("independence") requires the absence of unobserved instrument-outcome (PIV^{EA}-earnings) confounding. We address the independence assumption in three main ways.

The first two are detailed in the main body of the text and only briefly restated here. First, we relax the independence assumption by adding family fixed-effects in the sibling-MR model. This adjusts for shared, family-level PIV^{EA}-earnings confounding (e.g., population stratification, assortative mating, and dynastic effects) in the 2SLS estimate. As reported in the main text, the sibling-MR point estimates are slightly larger, statistically significant, and less precise than the main MR results (Figure 2, Table S2). The comparison of sibling-MR and MR results provides no evidence for a violation of the A2 independence assumption.

Second, we compute Norway-only models in which the PIV^{EA} is constructed from an FGWAS within siblings on Norwegian data. This removes family-level PIV^{EA}-schooling confounding, creates a plausibly exogenous PIV^{EA}, and hence remediates concerns about unobserved PIV^{EA}-earnings confounding. As reported in the main text, the Norway-only-MR point estimates are slightly larger, statistically significant, and less precise than the main MR results (Figure 2, Table S2). The comparison of Norway-only MR and MR results provides no evidence for a violation of the A2 independence assumption.

Third, although the A2 assumption can never fully be tested, a strong version can be falsified with covariate-balance tests (22). Specifically, under the strong version of the A2 assumption that the PIV^{EA} is not confounded by *any* covariate, the PIV^{EA} should be unassociated with the observed covariates. The covariate balance test thus regresses the PIV^{EA} on observed covariates to measure these associations. Since balance is an in-sample property, Imai, King and Stuart (23) argue that balance should be assessed by inspecting the observed magnitude of associations rather than evaluating statistical tests that relate sample associations to population parameters. We further note that only unobserved PIV^{EA}-earnings confounding that is large relative to the first stage would induce large bias in the MR estimates. For completeness, we assess violations of the A2 assumption by inspecting the magnitude of the observed associations and also by evaluating their joint statistical significance (using an F-test to avoid distortion from multiple testing).

We assessed covariate balance in the full genotyped MoBa sample, the sibling genotyped MoBa sample, and the Norway-only MoBa estimation sample (Figure S3).

Figure S3A for the full genotyped MoBa sample shows that the associations between the PIV^{EA} and observed covariates are substantively small (never exceeding 0.06 SD). The highest association indicated that one year increase in parents' years of schooling was related to a 0.057 SD (95% CI=0.054-0.060) higher PIV^{EA}, i.e., merely ≈ 2 percentile points (24). The joint *F*-test across all observed covariates is statistically significant ($F(8, 23) = 555.36$, $p < 0.0001$), likely owing to testing in a large sample (N=109,800).

Figure S3B for the MoBa genotyped siblings sample shows no indications of imbalance. All coefficients from a within-sibling regression of PIV^{EA} on observed covariates are tightly centered around zero (e.g., a one-year increase in parents' years of schooling was related to a 0.028 (95% CI=-0.024-0.08) increase in the PIV^{EA}). The joint *F*-test is not statistically significant ($F(7, 9092) = 0.92$, $p = 0.4895$, N=18,666).

Finally, Figure S3C for the Norway-only PIV^{EA} also shows negligible associations between the Norway-only PIV^{EA} and observed covariates (the association between parents' years of schooling and the PIV^{EA} was 0.02 SD (95% CI=0.018-0.026). The joint *F*-test across all observed covariates is statistically significant ($F(8, 23) = 97.79$, $p < 0.0001$), likely owing to a large sample (N=89,179).

In conclusion, the covariance balance tests provide no evidence of A2 independence violations for the sibling MR model, and none but evidence that appears trivial in magnitude for the full MR and Norway-only MR models. To adjust for any lingering A2 violations related to observed covariates, all MR models adjust for observed covariates.

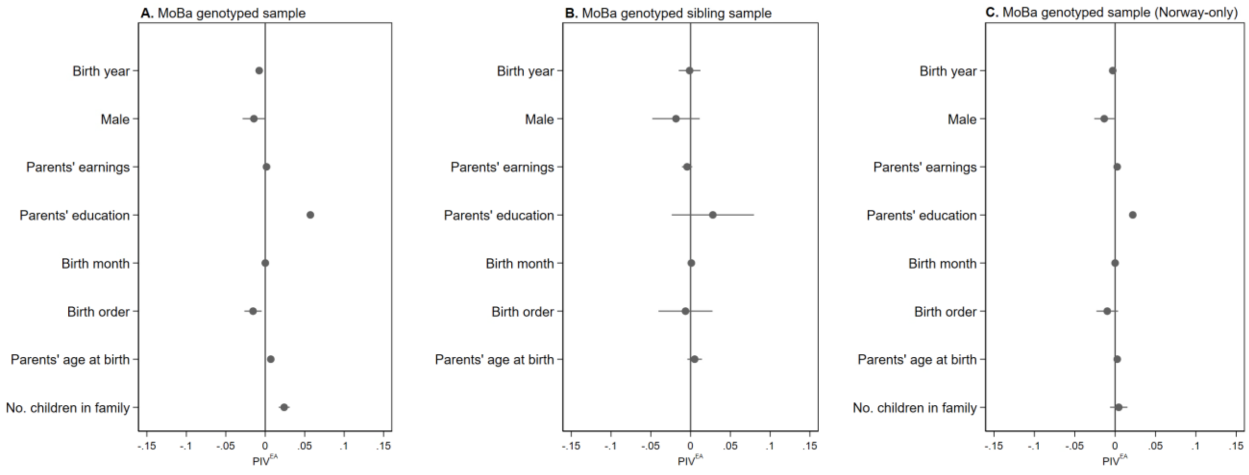

Figure S3. Assessment of covariate balance for the polygenic instrumental variable. In Panel A, we use the full genotyped MoBa sample (*N*=109,800) and regress standardized PIV^{EA} on covariates. In Panel B, we use the genotyped MoBa sibling sample (*N*=18,666) and regress standardized PIV^{EA} on covariates with sibling fixed-effects. Number of children in family is constant and thus not included in Panel B. In Panel C, we use the Norway-only genotyped sample (*N*=89,179) and regress the Norway-only standardized PIV^{EA} on covariates.

3.3.2 Exclusion (A3): Summary-level MR to test and adjust for pleiotropy

Assumption A3 (exclusion) of the conventional MR model using a PIV^{EA} requires that the genetic variants in the PIV^{EA} are associated with earnings only via the causal effect of EA on earnings. This assumption would be violated if genetic variants in the PIV^{EA} exerted direct effects on earnings (horizontal pleiotropy). If genetic variants exert both positive and negative direct effects on earnings that do not cancel each other out (directional pleiotropy, a special case of horizontal pleiotropy), the MR point estimate would be biased (25).

We assess the exclusion assumption in multiple and complementary ways. In this subsection, we test and adjust for exclusion violations by executing summary-level MR models that were specifically developed to test and adjust for various forms of pleiotropy under different auxiliary assumptions (25, 26). (See the next subsection 3.3.3 for a formal sensitivity analysis.)

Summary-level MR models take advantage of the basic fact that each SNP-specific MR estimate, β_G, can be expressed as a ratio of the reduced form coefficient from the regression of the outcome, earnings Y, on the genetic variant, G, $\beta_{Y,G}$, over the first-stage coefficient from the regression of the exposure, schooling

S, on the genetic variant, $\beta_{S,G}$, $\beta_G = \frac{\beta_{Y,G}}{\beta_{S,G}}$ (25). To avoid overfitting, the SNP-specific first-stage and reduced-form coefficients in summary-level MR should be obtained from independent samples (25). We use SNP-specific first-stage coefficients from the international data of the EA4 GWAS (12) (excluding MoBa and 23andMe participants) and SNP-specific reduced-form coefficients estimated from MoBa.[1] Summary-level MR models then aggregate the SNP-specific estimates, β_G, into an overall estimate for the returns to schooling.

As a baseline model, we present inverse-variance weighted MR (IVW-MR), which pools all SNP-specific MR estimates and their standard errors into a single weighted-average (25). Like conventional MR, IVW-MR requires the A3 exclusion assumption and is not robust to pleiotropy. If the first stage and the reduced form were estimated from the same population, IVW-MR and the conventional single-sample MR estimate should closely coincide. However, since the first stages and reduced forms in IVW-MR are estimated from different samples, IVW-MR and conventional MR estimates can differ (25), as they do in our analysis (see below). The presence of pleiotropy in our main MR analysis must thus be judged by comparing the pleiotropy-robust summary-level MR models against the baseline summary-level IVW-MR estimates presented in Figure S4, not against the main MR model with individual level data presented in Figure 2. Comparing the results of pleiotropy-robust summary-level MR results against the summary-level IVW-MR results does inform the presents of pleitropy in our main MR model, because pleiotropy affects MR estimates via distorting the reduced form, and the reduced forms of our IVW-MR and our main MR models are estimated from the same weights and genetic variants.

Next, we present results from multiple pleiotropy-robust models.

Results from summary-level MR analyses

Table S4 presents results from our five summary-level MR estimators. Figure S4 displays the same information as a coefficient plot. Figure S6 presents an MR-scatterplot with the reduced-form and first-stage values for each of the 335 SNPs in the PIV^{EA}, where the slopes of the fitted lines give the point-estimate for each estimator (25). Cochran's Q-statistic (Q=476, df=334, p<0.001) indicates heterogeneity across SNP-specific estimates for the returns to schooling. Under the additional assumption of constant

---

[1] We used the same 335 SNPs selected for the PIV^{EA}. We obtained SNP-earnings effects in the genotyped MoBa participants (N=109,800) and ran a GWAS using fastGWA with 20 principal components, year of birth, sex, and batches as covariates.

returns to schooling across the population (no effect heterogeneity), this could indicate horizontal (but not necessarily biasing directional) pleiotropy; alternatively, it could indicate effect heterogeneity in the returns to schooling.

IVW-MR

The baseline estimate for the returns to schooling from IVW-MR, against which all subsequent estimates in this subsection (3.3.2) should be compared was 0.049 (95% CI=0.045-0.053) log points. We note, again, that IVW-MR is not robust to pleiotropy.

MR-Egger

Unlike IVW-MR, MR-Egger is robust to directional pleiotropy under the additional assumption that the instrument strength is independent of the pleiotropic direct effects (InSIDE). The intercept of the MR-Egger model captures—and thus tests for—bias due to directional pleiotropy (25, 27), and the MR-Egger slope estimate recovers a pleiotropy-adjusted effect estimate. The MR-Egger intercept is very small and not statistically significant (α=-8.9×10^{-4}, 95% CI=−1.8×10^{-3}-1.0×10^{-6}). The returns to schooling estimate of the MR-Egger slope (log points=0.065, 95% CI=0.048-0.081) overlaps with the baseline IVW-MR estimate. MR-Egger thus fails to detect evidence of directional pleiotropy.

MR-Median, MR-Mode (weighted and simple), and MR-Corge

The MR-Median (weighted-median) estimator (28) assumes that at least 50% of the weight in the analysis comes from valid IVs (no horizontal pleiotropy). Thus, MR-Median permits that up to 50% of the weight in the analysis comes from invalid IVs. Our MR-Median estimate was 0.050 (95% CI=0.045-0.056) log points, which agrees with the IVW-MR baseline, providing no evidence of pleiotropy.

MR-Mode estimators (29) group the SNP-specific effects estimates into clusters and then report the estimate for the largest cluster (the "mode") as the causal effect. MR-Mode thus assumes that the SNPs in the largest cluster are valid and permits that all other SNPs are invalid due to horizontal pleiotropy (25, 26, 29). MR-Mode (weighted) adds precision weighting; MR-Mode (simple) does not. Our weighted MR-Mode estimate was 0.055 (95% CI=0.037-0.073) log points, and the simple MR-Mode estimate was 0.051 (95% CI=0.031-0.072) log points. Both estimates closely agree with the baseline IVW-MR estimate, providing no evidence of pleiotropy.

MR-Corge relies on the core gene hypothesis, which states that a small number of core genes have a direct impact on the trait and those core genetic variants are more likely to have stronger variant-trait

associations (21). By contrast, variants with smaller impacts on the trait are hypothesized to act through peripheral pathways that may also exert pleiotropic direct effects on the outcome. With MR-Corge, we rank our 335 genetic variances by SNP effect size and categorize them into k groups: SNPs categorized in the first group are more strongly associated with schooling and more likely to be core genetic variants. We conduct analyses with different values of k as sensitivity checks. We report results for k=35 (groups of about 10 SNPs) and for k=10 in Figure S7 and Table S5. Our putative core instruments (SNPs most strongly associated with schooling, in the first groups) had higher F-statistics than the F-statistics based on all instruments (with k=35, 1st group F=2,398 vs overall F=811). The returns to schooling estimated from these core instruments are statistically significant (e.g., with k=35, 1st-group 0.067 log points, 2nd-group 0.042 log points, p-values $< 1e-6$) and align with the baseline IVW-MR estimate. Moreover, using instruments that are less associated with schooling does not result in stronger estimates. This suggests that our MR returns estimates are not driven by less-associated, potentially more pleiotropic, SNPs.

Leave-one-out sensitivity analysis for MR-Egger

Figure S5 shows a leave-one-out sensitivity analysis for MR-Egger, which examines potential bias due to a single pleiotropic variant by iteratively removing one of the 335 SNPs from MR-Egger at a time (25). Large deviations from the mean would suggest SNP-specific pleiotropy (14). The results in Figure S5 show no such deviations and hence no evidence of SNP-specific pleiotropy.

Overall evaluation

These results show remarkable consistency across all five summary-level MR methods, with point estimates for the returns to schooling narrowly ranging from 0.049 to 0.067 log points. All confidence intervals exclude zero, indicating a statistically significant positive causal effect.

The convergence of estimates is reassuring. Despite some indication of heterogeneity across SNPs from Cochran's Q-test, the IVW estimate (0.049) aligns closely with the robust methods designed to handle invalid instruments under their respective auxiliary assumptions. The MR-Median estimate (0.050) and both MR-Mode estimates (0.051 and 0.055) are nearly identical to the baseline IVW estimate, and the MR-Egger estimate (0.065) and first- and second-group MR-Corge estimates (0.067 and 0.042) are in the same ballpark with wider confidence intervals. A non-significant MR-Egger intercept and close agreement across all summary-level methods fail to provide any evidence for bias due to pleiotropy in our main MR models.

No Norway-only summary-level analyses

As a robustness check to address portability concerns regarding the first-stage estimates from the international EA4 GWAS in the above summary-level MR models, we also attempted to perform analogous summary-level MR analyses using first-stage coefficients from our Norway-only FGWAS. Unfortunately, the first stage was too weak to proceed. With an *F*-statistics of only 11.7, estimates from Norway-only summary-level MR models would be attenuated towards zero (30) and both *t*-ratio inference and AR inference for the returns to schooling would be incorrect (19).

	Log (Earnings)	
Two-sample MR estimator	Coef.	SE
IVW-MR	0.049***	(0.002)
MR-Egger	0.065***	(0.008)
MR-Median	0.050***	(0.003)
MR-Mode (weighted)	0.055***	(0.009)
MR-Mode (simple)	0.051***	(0.011)

Table S4. Returns to schooling estimates from pleiotropy-robust two-sample Mendelian randomization estimators. Standard error in parentheses. For visual presentation, see Figure S4. *** $p < 0.001$, ** $p < 0.01$, * $p < 0.05$.

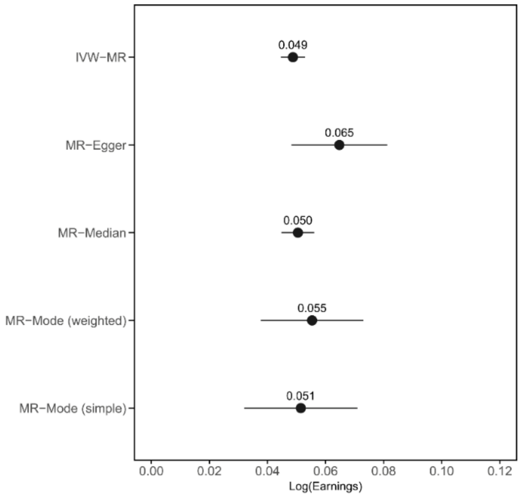

Figure S4. Returns to schooling estimates from pleiotropy-robust two-sample Mendelian randomization estimators. Estimates with 95% confidence intervals from the following estimators: inverse-variance weighted MR (IVW-MR), MR-Egger, MR-Median, MR-Mode (weighted), MR-Mode (simple).

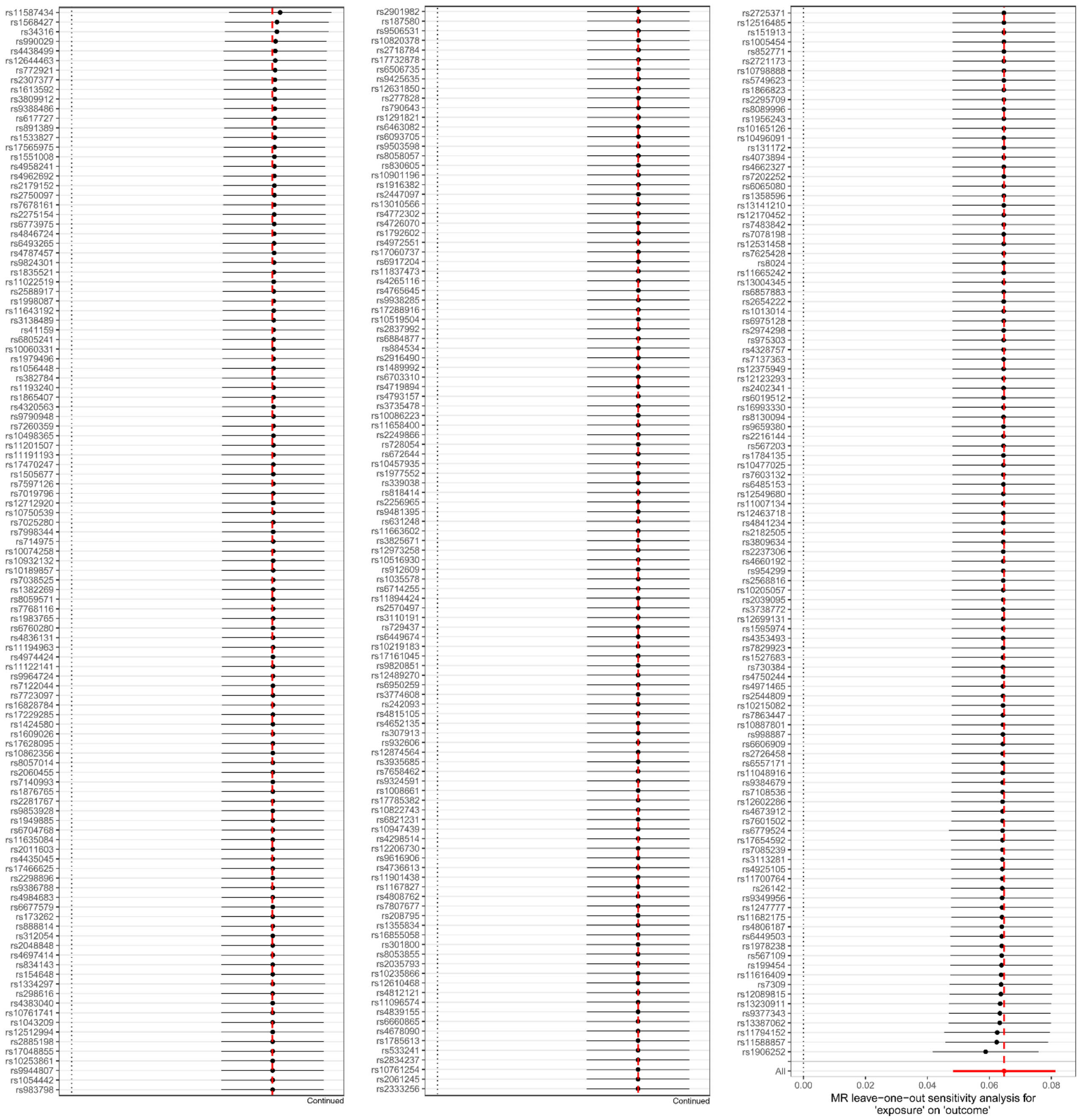

Figure S5. Leave-one-out sensitivity analysis for MR-Egger. The vertical black dotted line on the *x*-axis shows the effect size of zero. The vertical red dotted line shows the overall MR-Egger estimate. The impact of removing any single SNP on the overall estimate can be seen by comparing the effect size with the specific SNP removed to this overall estimate.

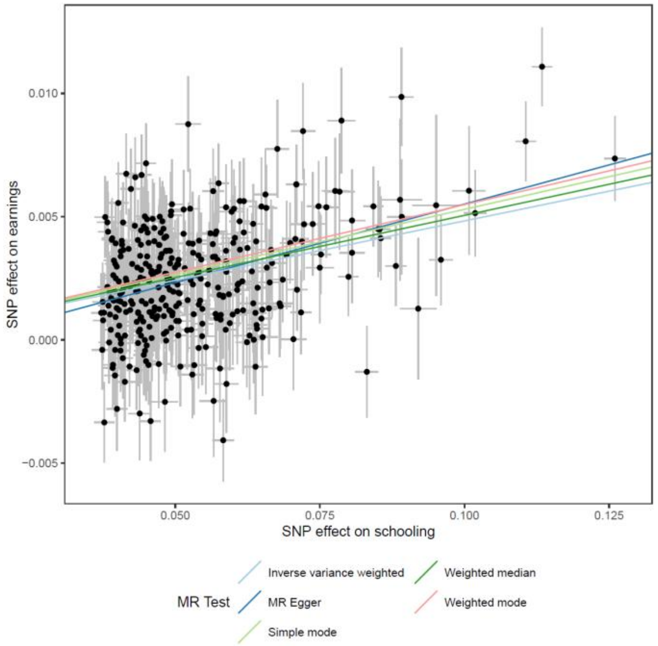

Figure S6. Two-sample Mendelian randomization scatterplot with pleiotropy-robust estimators. SNP-schooling (*x*-axis) and SNP-earnings (*y*-axis) associations and 95% confidence intervals plotted per SNP. Lines represent fitted values from the estimators inverse-variance weighted (IVW-MR), MR-Egger, MR-Median (weighted median), and MR-Mode (weighted mode and simple mode). Cochran's *Q*-statistic for IVW-MR was Q=476, df=334, p<0.001.

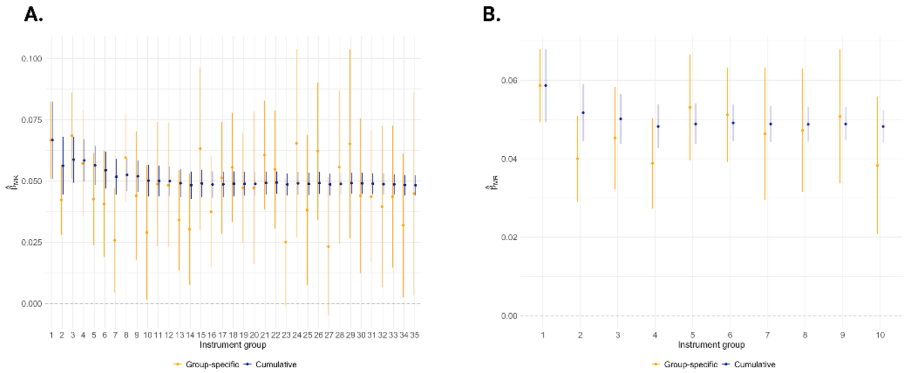

Figure S7. Two-sample MR-Corge IVW estimates. Panel A presents results with k=35; Panel B presents results with k=10.

	Group	Number of SNPs in the group	*F*-statistic	IVW estimate	SE	*p*-value
k=35	1	10	2398.106	0.067	0.008	0.000
	2	10	1817.252	0.042	0.007	0.000
	3	9	1160.169	0.068	0.009	0.000
	4	10	1393.894	0.057	0.011	0.000
	5	9	1083.325	0.043	0.010	0.000
	6	10	1101.824	0.041	0.011	0.000
	7	9	983.949	0.026	0.011	0.018
	8	10	1017.689	0.059	0.009	0.000
	9	9	806.642	0.044	0.013	0.001
	10	10	981.806	0.029	0.014	0.039
	11	9	851.784	0.049	0.013	0.000
	12	10	763.636	0.048	0.013	0.000
	13	10	796.002	0.034	0.010	0.001
	14	9	733.766	0.030	0.012	0.009
	15	10	770.802	0.063	0.017	0.000
	16	9	718.083	0.037	0.012	0.001
	17	10	641.887	0.051	0.012	0.000
	18	9	743.616	0.056	0.011	0.000
	19	10	662.478	0.047	0.011	0.000
	20	9	647.248	0.047	0.016	0.003
	21	10	659.612	0.061	0.011	0.000
	22	9	634.458	0.055	0.012	0.000
	23	10	658.346	0.025	0.013	0.058
	24	10	620.066	0.065	0.020	0.001
	25	9	605.885	0.038	0.016	0.015
	26	10	615.453	0.062	0.014	0.000
	27	9	558.139	0.023	0.014	0.106
	28	10	568.116	0.056	0.016	0.000
	29	9	565.614	0.065	0.020	0.001
	30	10	545.982	0.044	0.016	0.006
	31	9	513.776	0.044	0.014	0.002
	32	10	502.975	0.039	0.017	0.019
	33	9	503.426	0.044	0.015	0.003
	34	10	492.576	0.032	0.015	0.033
	35	10	469.547	0.045	0.021	0.034
k=10	1	34	1875.394	0.059	0.005	0.000
	2	33	1124.053	0.040	0.006	0.000
	3	34	960.821	0.045	0.007	0.000
	4	33	788.797	0.039	0.006	0.000
	5	33	732.107	0.053	0.007	0.000
	6	34	682.058	0.051	0.006	0.000
	7	33	643.057	0.046	0.009	0.000
	8	34	599.267	0.047	0.008	0.000
	9	33	543.067	0.051	0.009	0.000
	10	34	499.819	0.038	0.009	0.000

Table S5. Two-sample MR-Corge IVW estimates with *k*=35 and *k*=10 for each group of instruments (i.e. group of SNPs ranked by the size of their first stage association with schooling).

3.3.3 Exclusion (A3): Sensitivity analysis

The union of confidence intervals (UCI; also known as "plausibly exogenous") sensitivity analysis examines the robustness of MR estimates to violations of the strict A3 exclusion assumption that the PIV^{EA} exerts zero direct effects on earnings. It does so by examining how strong a hypothetical direct effect of the PIV^{EA} on earnings would have to be to alter the conclusion of our main MR estimate (Figure 2) (31).

The UCI approach considers a structural equation for earnings,

$$Y_i = \alpha + \beta S_i + \gamma Z_i + X_i'\pi + \varepsilon_i, \qquad (10)$$

that includes a direct (e.g., pleiotropic) effect of the PIV^{EA}, Z, on earnings, γ. Exclusion holds if $\gamma = 0$. With a single IV, the exclusion assumption cannot be tested empirically by regressing Y on $S, Z,$ and X due to collider bias (32): adjusting for S would induce a spurious association between Z and Y through U (i.e., path: $Z \rightarrow \boxed{S} \leftarrow U \rightarrow Y$) that would yield an estimated $\hat{\gamma} \neq 0$ even if exclusion holds, $\gamma = 0$, as illustrated in Figure S8.

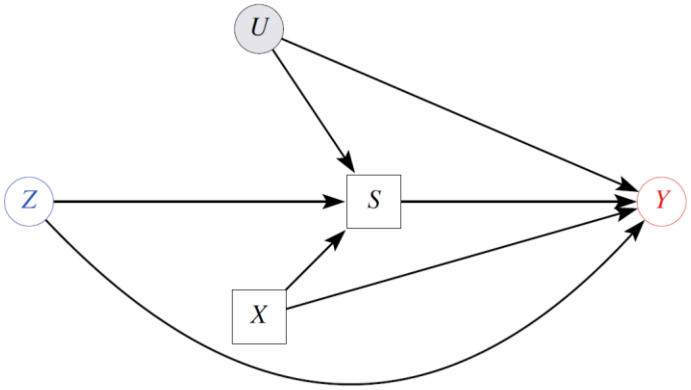

Figure S8. Causal graph for collider bias. Directed acyclic graph for the association between PIV^{EA}, Z, and earnings, Y, adjusted for years of schooling, S, and other observed covariates, $\boldsymbol{X}$. U are unobserved $S - Y$ confounders. Figure created using Causal Fusion (causalfusion.net).

The UCI sensitivity analysis does not aim to test exclusion by estimating γ but rather adjusts MR estimates for a range of hypothetical values of γ. The exclusion-corrected MR estimate for a specific hypothetical exclusion violation is obtained by subtracting the assumed value of the direct effect of Z on Y, γ, divided by the first stage, $Cov(S, Z)/V(Z)$, from the conventional MR estimate (31, 33):

$$\beta(\gamma) = \frac{Cov(Y_i,\ Z_i)}{Cov(S_i,\ Z_i)} - \frac{\gamma}{\frac{Cov(S_i, Z_i)}{V(Z_i)}} \tag{11}$$

UCI computes a 95% confidence interval for each assumed direct effect and then reports the upper and lower bounds of the union of these CIs across a range of assumed direct effects. We apply this sensitivity analysis to MR in the full genotyped MoBa sample, using the "plausexog" command by Clarke and Matta (34).

Figure S9 reports the confidence bounds on exclusion-corrected MR estimates for the return to schooling under a range of hypothetical exclusion violations, ranging from no exclusion ($\gamma = 0$ NOK) up to a direct causal effect of PIV^{EA} on earnings of $\gamma = 20{,}000$ NOK.

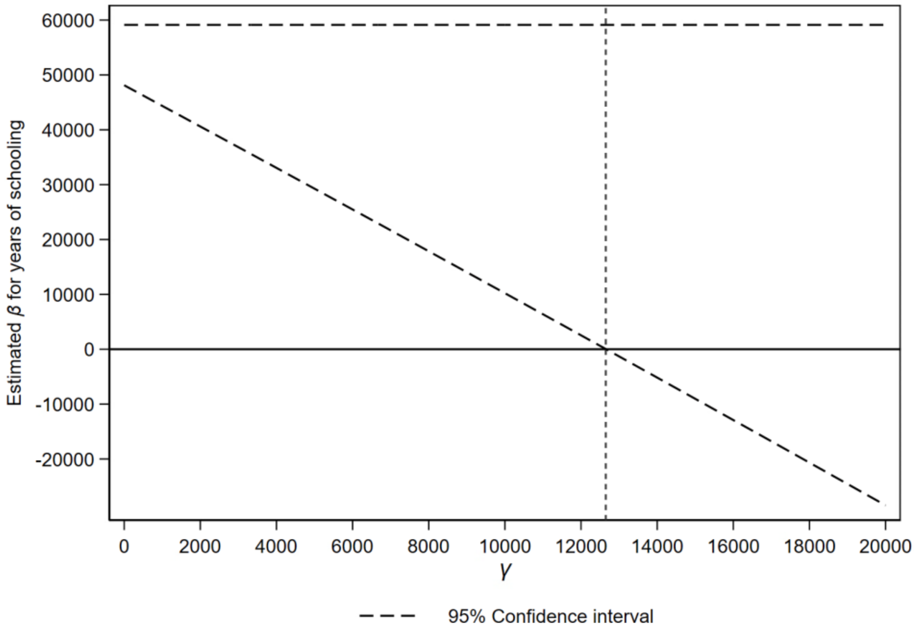

Figure S9. Results from union of confidence intervals sensitivity analysis for MR. The upper and lower dashed lines give the upper and lower confidence bounds for the exclusion-corrected MR estimates for direct effects of the PIV^{EA} on earnings ranging from 0 to γ at each γ. Implemented using "plausexog" (31).

Figure S9 shows that our MR estimate for the returns to schooling would become statistically insignificant (the lower bound of the UCI would drop below 0) if the pleiotropic direct effect of the PIV^{EA} on earnings exceeded NOK 12,650.

This would constitute a very large exclusion violation. Considering that the reduced form of the main MR model presented in Figure 2, Panel B (the regression of earnings on PIV^{EA} and covariates) gives an overall

association between a one-unit increase in the PIV^{EA} and earnings of NOK=14,133 (95% CI=12,227-16,039), the exclusion violation would have to amount to 89.5% (12,650/14,133) of the reduced form to reduce our MR estimate to statistical insignificance. In other words, in order to discount the MR-based evidence that schooling positively affects earnings, one would have to argue that almost all of the association between the PIV^{EA} and earnings, net of covariates, goes through pathways other than EA. This seems unlikely, since the PIV^{EA} was specifically developed to predict EA.

In sum, this sensitivity analysis supports statistically significant positive returns to schooling even for exclusion violations up to 89.5% of the reduced form estimate.

3.3.4 Monotonicity (A4) and MR-OLS decomposition

Recall that OLS and MR provide different estimates for the returns to schooling (Figure 2 and Table S2). This subsection investigates the difference between the OLS and MR point estimates for the returns to schooling using the IV-OLS (henceforth MR-OLS) decomposition method (35). One part of this decomposition also provides a falsification test for the A4 monotonicity assumption.

We execute the MR-OLS decomposition on the MoBa genotyped sample, with categorized covariates (as required by available software). The baseline estimates for OLS and MR models in this approach (Table S6) closely align with our main estimates (Figure 2).

	Log(Earnings)	SE	% of MR-OLS gap
OLS	0.052***	(0.001)	
MR	0.076***	(0.004)	
MR-OLS Gap	0.024***	(0.004)	
Covariate weight	0.001*	(0.001)	4.17%
Treatment-level weight	0.003***	(0.001)	12.50%
Marginal effect	0.020***	(0.004)	83.33%

Table S6. MR-OLS decomposition. Analyses based on the MoBa genotyped sample (N=109,800) including the same covariates as main specifications (Figure 2). Robust standard errors (SE) in parentheses. *** $p < 0.001$, ** $p < 0.01$, * $p < 0.05$.

The MR-OLS decomposition separates the MR-OLS difference into three components: differences due to covariates (effect heterogeneity; different characteristics among compliers and the general population), treatment-levels (non-linearity; differences in schooling levels), and marginal effects (capturing unobserved confounding and/or different effects between compliers and the general population; the latter components are not separable in this decomposition). The sum of the weights of the decomposition is scaled to one across levels of schooling and, separately, within groups defined by covariates (e.g., across parents' educational level).

We begin by summarizing the three main results of the decomposition. First, covariate weight differences only account for 4.2% of the MR-OLS gap (Table S6). Table S7 goes into detail: MR estimates tend to place higher weights on disadvantaged individuals, particularly those from families with lower income and lower education. Individuals in the lowest family income quartile received a weight of 0.30 in MR compared to 0.27 in OLS, while those from families with low parental education had weights of 0.70 in OLS compared to 0.73 in MR. Second, treatment-level weight differences account for 12.5% of the MR-OLS gap (Table S6). Figure S10 shows that MR puts similar weights to OLS on levels of education, with slightly higher weight on master's degrees; 0.07 in OLS and 0.09 in MR. Third, most of the MR-OLS gap (83.3%) comes from the marginal effect component (Table S6). This suggests that most of the MR-OLS gap stems from a combination of (a) MR-OLS differences in accounting for unobserved confounding and/or (b) compliers in MR having stronger returns to schooling compared to the general population.

Next, we provide more details on each decomposition component.

Treatment level weights. From Figure S10, we see that OLS and MR put very similar weights on most education levels, with both putting most weight on entering the vocational track (from 13 years) to

obtaining a bachelor's degree (16 years). MR, however, puts a little more weight on master's degree (17-18 years). This finding agrees with the MR analysis of Davies, Dickson, Davey Smith, Windmeijer and van den Berg (36), who compared the extent to which the polygenic index for educational attainment impacted the probability of remaining in school by age versus their school-reform (raising of school leaving age) IV. The latter had a local impact reducing school leaving prior to age 16. Comparing our MR weights to analyses of school-reform IV weights in Norway (6), school-reform IV estimates are local to the specific year raised by the reform, while our MR weights indicate that the MR estimates are not local to a specific year, but rather concern a general additional year of schooling almost exactly like OLS.

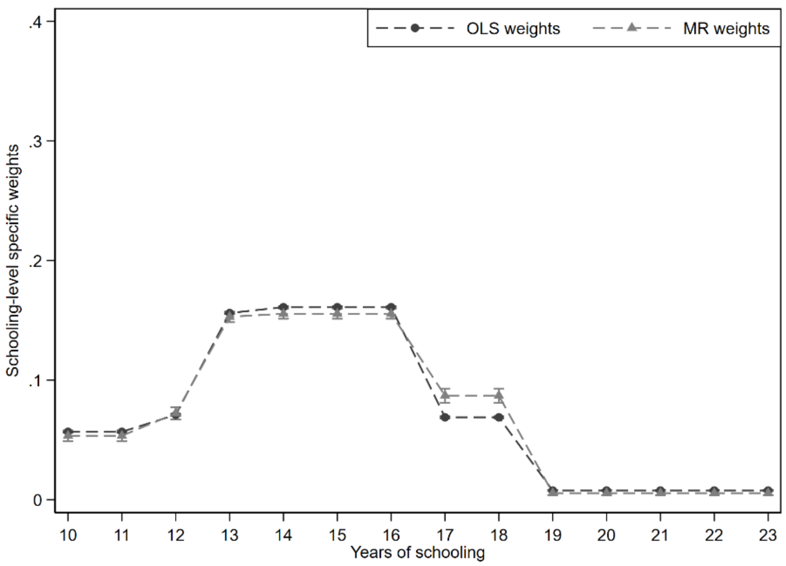

Figure S10. Treatment-level specific weights for MR and OLS. We report treatment-level specific weights for MR and OLS with 95% confidence intervals, including the same covariates as in the main specifications (Figure 2). Weights sum to 1 for MR and OLS separately. Weights outside observed years of schooling are zero with this weighting approach and are not shown. Details on weight construction in Ishimaru (35).

Variable	Group	Group share	OLS weight	MR weight	MR-OLS weight differences	Subsample returns to schooling coefficient
Family earnings	1st quartile	0.251	0.266 (0.002)	0.302 (0.012)	+0.036	0.057 (0.001)
	2nd quartile	0.247	0.253 (0.002)	0.256 (0.012)	+0.003	0.053 (0.001)
	3rd quartile	0.248	0.245 (0.002)	0.226 (0.011)	-0.019	0.049 (0.001)
	4th quartile	0.253	0.236 (0.002)	0.217 (0.011)	-0.019	0.049 (0.001)
Family education	Low (< 12 yrs.)	0.644	0.697 (0.002)	0.728 (0.012)	+0.031	0.054 (0.001)
	Medium (≥ 12-15 yrs.)	0.235	0.218 (0.002)	0.188 (0.011)	-0.030	0.051 (0.001)
	High (≥ 16 yrs.)	0.120	0.086 (0.002)	0.085 (0.007)	-0.001	0.052 (0.002)
Sex	Men	0.400	0.564 (0.003)	0.581 (0.013)	+0.017	0.041 (0.001)
	Women	0.600	0.436 (0.003)	0.419 (0.013)	-0.017	0.067 (0.001)
Cohort	1959-1964	0.043	0.055 (0.001)	0.058 (0.006)	+0.003	0.051 (0.002)
	1965-1970	0.235	0.252 (0.002)	0.266 (0.012)	+0.014	0.055 (0.001)
	1971-1976	0.458	0.430 (0.002)	0.420 (0.013)	-0.010	0.054 (0.001)
	1977-1982	0.264	0.263 (0.002)	0.256 (0.012)	-0.007	0.059 (0.001)
Birth month	1st quarter	0.254	0.252 (0.002)	0.224 (0.012)	-0.028	0.057 (0.001)
	2nd quarter	0.270	0.265 (0.002)	0.281 (0.012)	+0.016	0.056 (0.001)
	3rd quarter	0.247	0.250 (0.002)	0.241 (0.012)	-0.009	0.055 (0.001)
	4th quarter	0.230	0.233 (0.002)	0.254 (0.011)	+0.021	0.055 (0.001)
Birth order	1	0.501	0.507 (0.002)	0.504 (0.013)	-0.003	0.057 (0.001)
	2	0.335	0.332 (0.002)	0.304 (0.013)	-0.028	0.054 (0.001)
	3	0.122	0.119 (0.002)	0.146 (0.009)	+0.027	0.053 (0.001)
	4	0.030	0.031 (0.001)	0.033 (0.005)	+0.002	0.054 (0.002)
	5	0.011	0.010 (0.001)	0.013 (0.003)	+0.003	0.052 (0.004)
Number of children in family	1	0.189	0.202 (0.002)	0.201 (0.011)	-0.001	0.061 (0.001)
	2	0.470	0.464 (0.003)	0.432 (0.013)	-0.032	0.054 (0.001)
	3	0.250	0.242 (0.002)	0.273 (0.012)	+0.031	0.055 (0.001)
	4	0.067	0.067 (0.001)	0.067 (0.007)	0.000	0.052 (0.002)
	5	0.024	0.025 (0.001)	0.027 (0.004)	+0.002	0.051 (0.003)
Parents age at birth	≤19	0.025	0.027 (0.001)	0.027 (0.001)	0.000	0.057 (0.003)
	20-24	0.282	0.296 (0.002)	0.290 (0.012)	-0.006	0.055 (0.001)
	25-29	0.381	0.365 (0.002)	0.346 (0.013)	-0.019	0.056 (0.001)
	30-34	0.196	0.193 (0.002)	0.202 (0.011)	+0.009	0.056 (0.001)
	35-39	0.082	0.083 (0.001)	0.088 (0.007)	+0.005	0.055 (0.001)
	≥40	0.034	0.036 (0.001)	0.047 (0.005)	+0.011	0.054 (0.002)

Table S7. MR and OLS weights on covariates groups. Results from MR-OLS decomposition applied to the full MoBa genotyped sample. Weights sum to one for each group. OLS-based subsample returns to schooling estimated by regressing log earnings on years of schooling with sex and birth cohorts as controls, and with robust standard errors in parentheses.

Covariate weights. Table S7 shows that there are no covariate-group-specific negative MR weights. The absence of negative MR weights supports the “monotonicity” assumption (A4) by failing to detect evidence of the existence of defiers (35).

In addition, Table S7 shows that the MR estimates put more weight on disadvantaged groups compared to OLS across the main differences we found. The strongest difference was for family earnings quartiles, where the lowest quartile received more weight in MR (+0.036) compared to OLS. Similarly, individuals from families with low parental education and with three children receive higher weight in MR (+0.031).

As the magnitude of differences across covariate weights and schooling-level weights is small, we are hesitant to read too much into them.

3.4 Additional analyses

3.4.1 Effect heterogeneity

	Log(Earnings)		Earnings (NOK)	
	OLS	MR	OLS	MR
Panel A: Sex				
Men	0.038***	0.048***	31,086***	44,019***
	(0.001)	(0.007)	(675)	(5,445)
Women	0.062***	0.100***	34,833***	61,180***
	(0.001)	(0.005)	(371)	(2,551)
Panel B: Family earnings				
1st quartile (lowest)	0.056***	0.078***	33,264***	47,665***
	(0.001)	(0.007)	(559)	(5,429)
2nd quartile	0.052***	0.081***	33,173***	55,795***
	(0.001)	(0.008)	(589)	(5,763)
3rd quartile	0.049***	0.075***	32,341***	52,656***
	(0.001)	(0.007)	(478)	(4,598)
4th quartile (highest)	0.048***	0.077***	33,891***	62,302***
	(0.001)	(0.009)	(965)	(7,094)

Table S8. Returns to schooling estimates by sex and family earnings. Each line refers to a separate, group-specific, model within the full genotyped MoBa sample (N=109,800). OLS and MR use the same covariates as our main specifications (Figure 2). Family earnings are measured in quartiles. For MR, the first stage F-statistic was strong in all models (Kleibergen-Paap rk Wald F-statistic for men: 338.8; for women: 1,213.9; for family-earnings: 220.6 to 513.7). Robust standard errors reported in parentheses. OLS uses robust t-tests and MR uses AR-tests. *** $p < 0.001$, ** $p < 0.01$, * $p < 0.05$.

Table S8 presents evidence on heterogeneity in the returns to schooling in the OLS and MR models (estimated on the same full genotyped MoBa sample). Both OLS and MR models found larger returns to schooling among women compared to men. In terms of log earnings, OLS finds somewhat higher returns to schooling among individuals from lower parental earnings quartiles, whereas MR does not. In terms of unlogged earnings, OLS indicates equal returns and MR suggests increasing returns across parental earnings quartiles.

3.4.2 Labor market experience

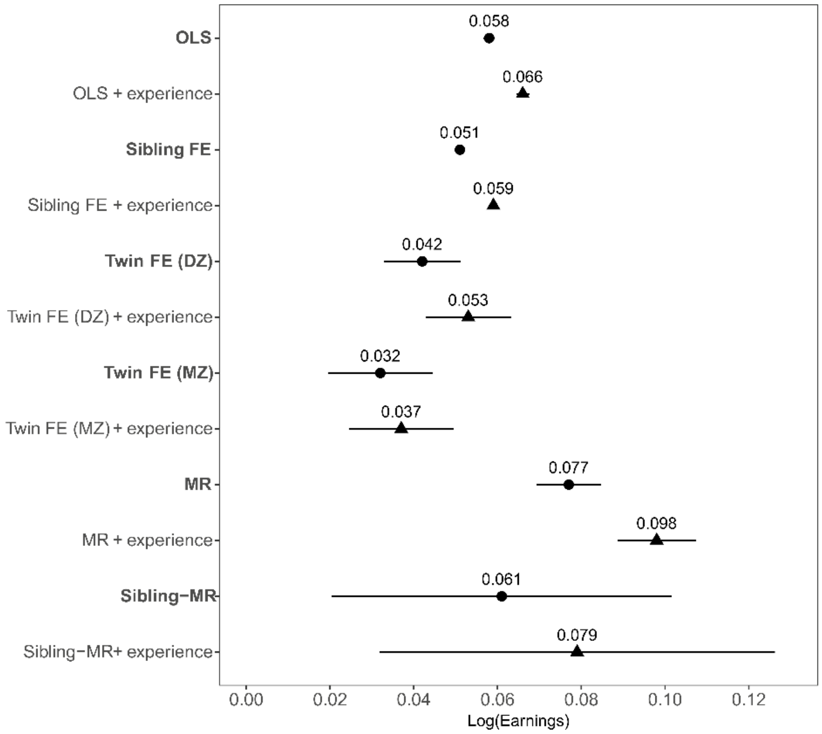

Figure S11. Returns to schooling with and without adjustment for years of labor market experience. We compare our main specification presented in Figure 2 (bold) using the same samples and models adding years of experience and years of experience squared to the adjustment set.

Figure S11 compares estimates from models with and without adjusting for years of labor market experience. Years of experience is commonly included in the Mincer equation for the returns to schooling (37), yet represents a "bad control" in causal analysis in that it is causally downstream from schooling (i.e., endogenous) and likely to introduce post-treatment bias (38, 39). Therefore, years of experience is not included in our main specifications (Figure 2).

Our measure of years of experience follows Eika and Kirkebøen (7): one year of full experience is assigned for each year of annual earnings above NOK 200,000, with earnings between 100,000-200,000 assigned experience from 0.5 to 1 as a linear function of earnings. Years of experience was measured with earnings between ages 17 to 33, thereby accounting for potential labor market experience obtained while in school (6). Thus, the years of experience measure captures experience while in school and post-school until age 33. Following conventional practice (37), we add years of experience and its squared term to our adjustment set.

Figure S11 shows that adding years of experience yields somewhat higher estimates across models (about 0.01 log points in OLS and about 0.02 log points in MR). The higher estimates reflects that schooling and experience are negatively correlated (38) (i.e., individuals with less schooling have accumulated more experience that raises their earnings). Adjusting for experience removes differences in experience, changes the estimand from a total to a direct effect of schooling, and may additionally introduce post-treatment bias (e.g., collider bias by unobserved experience-earnings confounding) (32, 38). Consequently, great caution is advised when interpreting models that include years of experience.

3.5 Life-cycle earnings and the internal rate of return

3.5.1 Age-specific returns to schooling and the internal rate of return (IRR)

Following Bhuller, Mogstad and Salvanes (6), we estimate age-specific returns to schooling, lifetime returns to schooling, and the internal rate of return (IRR). Here, we provide a brief description along with our implementation.

Returns to schooling is conventionally obtained by the Mincer model (6, 40) as shown in Equation 2,

$$log(Y_i) = \beta_0 + \beta_1 S_i + X_i' \beta_2 + \varepsilon_i \tag{2}$$

where, β_1 represents the change in log earnings associated with one additional year of schooling. This parameter is typically interpreted as the earnings growth rate by years of schooling (6, 41, 42).

By contrast, the IRR is the rate of return to additional schooling found by equating life-cycle costs (e.g., forgone earnings while in school) and life-cycle benefits (higher post-schooling earnings) when both are converted to their present value. For example, an IRR of 10% means that investing in additional schooling yields a 10% annual return, accounting for costs and benefits across the life-cycle. If alternative investments of foregone earnings yield less than 10% annual returns, education would be the more profitable choice.

We computed the IRR following Bhuller, Mogstad and Salvanes (6). Their approach has three advantages. First, it captures the life-cycle patterns of returns to schooling by separately estimating education effects at each age. Second, it allows earnings profiles to vary by education level. Third, it uses these age-specific returns to compute the IRR.

We compute the IRR in two steps, here described for one cohort followed from age 17 to 62, without covariates:

First, we compute the age-specific returns to education. For example, the OLS estimate is obtained by running

$$Y_{ai} = \alpha_a + \beta_a S_i + \varepsilon_{ai}, \tag{13}$$

where Y_a is annual earnings at age a, α_a is the intercept, β_a is the increase in annual earnings with one additional year of schooling, S is years of schooling, and ε_a is the error term. Our fixed-effects and MR estimates for the IRR are based on fixed-effects and MR analogues of this equation.

Second, we compute the IRR as the "discount rate (ρ) that equates the present value of potential income streams for different schooling levels" (6) as the solution to equation 14.

$$\sum_{a=17}^{62} \frac{\beta_a}{(1+\rho)^{a-16}} = 0 \tag{14}$$

The profitability of education is then estimated by comparing the IRR, ρ, to opportunity costs, which is proxied by the real market interest rate, r (2.3% in Norway, 1967-2010) (43).

We obtain two measures of the lifetime profitability of an additional year of schooling (6). First, equation 15 gives the undiscounted lifetime mean of the age-specific premium to an additional year of schooling:

$$\bar{\beta} = \sum_{a=17}^{62} \frac{\beta_a}{62-16} \tag{15}$$

Alternatively, the education premium can be expressed as the annuity of lifetime earnings, which discounts the age specific returns to education to their present value. This allows a direct comparison with alternative investments with constant returns,

$$\tilde{\beta} = \tilde{r} \sum_{a=17}^{62} \frac{\beta_a}{(1+r)^{a-16}} \tag{16}$$

where, $\tilde{r}$ denotes the annuity constant (i.e., $\tilde{r} = r/[1-(1+r)^{-(62-16)}]$) that converts the lifetime discounted premium into an annual gain. Annuity calculations are typically lower than education premiums in lifetime earnings as earning premiums toward the end of the working career are considerably discounted (6).

3.5.2 Data for lifetime returns to schooling analyses

We use the same samples as in our main specifications of Figure 2. We estimate returns to schooling using OLS, sibling fixed-effects, twin fixed-effects (for statistical power, we combine DZ and MZ twins), and MR. We followed the approach of Bhuller, Mogstad and Salvanes (6) and consequently implement the following restrictions: Birth municipality is used as childhood municipality (if missing, this was replaced by mother's or father's municipality of residence when the individual was born). Samples are restricted to individuals with municipality data since their (6) models include municipality and birth cohort fixed-effects and standard errors are two-way clustered by birth cohort and municipality. We used earnings data unrestricted at bottom or top (6). Years of schooling is the highest level attained for the individual. These restrictions imply only minor changes to sample sizes (Table S9). All our analyses are conducted at the individual level. Bhuller, Mogstad and Salvanes (6) used cohort-municipality-level data for OLS and IV. Our results are substantively identical regardless of using individual- or birth cohort-municipality level data for OLS and MR.

3.5.3 Lifetime returns to schooling and IRR

	OLS	Sibling FE	Twin FE	MR
Panel A: Education premiums				
Mean earnings, 17-24	-15,335***	-12,138***	-12,558***	-23,444***
	(439)	(61)	(722)	(1,467)
	[-0.072]	[-0.057]	[-0.065]	[-0.128]
Mean earnings, 25-44	17,986***	10,451***	5,905***	34,021***
	(414)	(127)	(1,703)	(2,823)
	[0.033]	[0.029]	[0.010]	[0.058]
Mean earnings, 45-62	45,420***	33,981***	28,605***	72,083***
	(518)	(238)	(3,043)	(4,290)
	[0.070]	[0.052]	[0.041]	[0.096]
Panel B: Lifetime education premiums				
Mean lifetime earnings	13,411***	8,403***	5,542***	21,544***
	(1,053)	(112)	(1,457)	(2,227)
	[0.028]	[0.017]	[0.011]	[0.043]
Annuity lifetime earnings	6,058***	3,341***	1,542*	10,336***
	(579)	(68)	(872)	(1,402)
	[0.020]	[0.011]	[0.005]	[0.033]
Panel C: Internal rate of return	0.100***	0.085***	0.068***	0.101***
	(0.001)	(0.001)	(0.008)	(0.003)
N	1,194,095	905,846	5,601	107,392

Table S9. Lifetime returns to schooling. We estimate returns to schooling for ages 17-62, and for ages 17-52 for MR due to availability of genotyped data from MoBa sampled 2002-2008 in our data. Earnings estimates are reported in NOK. Estimated coefficients scaled by mean earnings (proportional change) reported in brackets. Standard errors are two-way clustered by birth cohort and municipality and reported in parentheses. All models include fixed-effects for birth cohorts and childhood municipality. The IRR is reported in decimal form with bootstrap-based standard errors using 250 replications. OLS=Ordinary least squares; FE=Fixed-effects; MR=Mendelian randomization.
*** $p < 0.001$, ** $p < 0.01$, * $p < 0.05$.

Table S9 presents our detailed results. See also Results in the main body of the text for a description and Figure 3 for a visual representation of the estimated age-earnings profiles. For comparison, using OLS, Bhuller, Mogstad and Salvanes (6) found a 5.8% increase in lifetime earnings (the increase in annual earnings averaged over the life-cycle), while their IRR estimate was 9.3% for men with earnings data for ages 17-62 for birth cohorts 1943-1963. For IV (using a school reform increasing mandatory schooling from 8 to 9 years and differential roll-out throughout Norwegian municipalities), their corresponding estimates are 2.0% for lifetime earnings and an IRR of 11.2%, respectively. The IRR is higher than the lifetime earnings increase as this accounts for the timing of costs and benefits. Costs (foregone earnings) come early, while benefits (higher earnings) last many years.

3.5.4 Sensitivity analysis: Balanced panel

Figure S12 shows similar patterns in estimates across designs as Figure 3, but with balanced panel data. While the use of unbalanced panels could introduce bias, we find similar results when restricting our focus to ages and birth cohorts for which we have balanced panel data (i.e., birth cohorts 1959-1967 for ages 17-55; birth cohorts 1959-1970 for ages 17-52). The corresponding IRR estimates from the balanced panel are similar yet less precise compared to the estimates from the unbalanced panel (OLS: 0.098 [SE=0.001]; sibling fixed-effects: 0.087 [SE=0.001]; twin fixed-effects: 0.054 [SE=0.020]; MR: 0.092 [SE=0.005]).

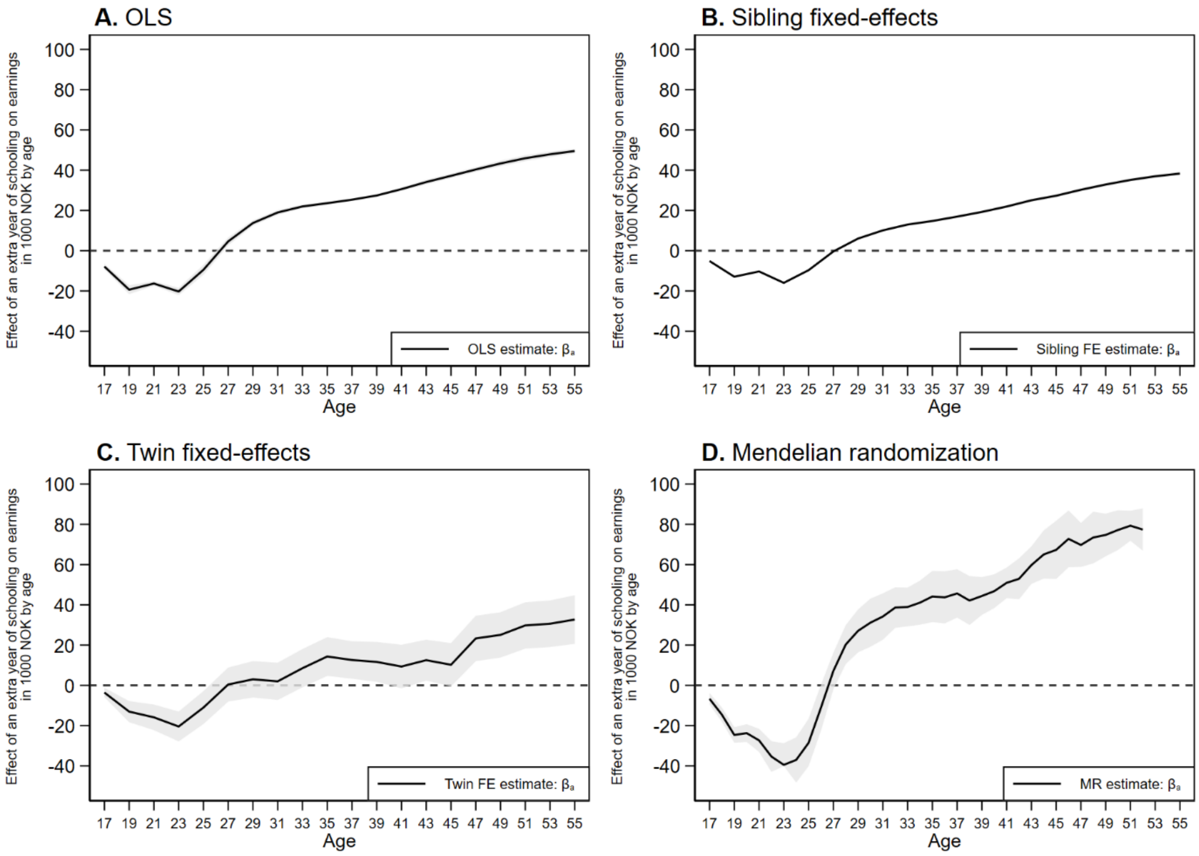

Figure S12. Estimates of age-specific returns to schooling with balanced panel. We report point estimates and 95% confidence intervals from the following models OLS, sibling fixed-effects, twin fixed-effects, and MR. Standard errors are clustered by cohort and childhood municipality. 95% confidence intervals represented by shaded areas. OLS=Ordinary least squares; FE=Fixed-effects. Earnings reported in 1000s of Norwegian kroners (NOK), wage-inflation adjusted to 2022 levels (USD/NOK ≈ 9.6).

4. Statistical software

Data preparation, individual-level analyses, and data visualization was conducted in Stata MP 18 (44), using reghdfe (45) and ivreghdfe (46), plausexog (31) for the UCI sensitivity analysis, ivolsdec (35) for MR-OLS decomposition, and weakiv for AR-test and 95% confidence intervals (47). Two-sample MR data preparation, analyses, and data visualization were conducted in R v. 4.2.3 (48), using the TwoSampleMR package (14). Plink 1.9 (49) was used to create PIV^{EA} and fastGWA (50) was used to obtain SNP-earnings effects.

5. References

1. Ministry of Health and Care Services (2018) Regulation on population-based health surveys (Forskrift om befolkningsbaserte helseundersøkelser). FOR-2018-04-27-645 (https://lovdata.no/dokument/SF/forskrift/2018-04-27-645).
2. P. Magnus *et al.*, Cohort profile: the Norwegian mother and child cohort study (MoBa). *International Journal of Epidemiology* **35**, 1146-1150 (2006).
3. R. E. Brandlistuen *et al.*, Cohort Profile Update: The Norwegian Mother, Father and Child Cohort (MoBa). *International Journal of Epidemiology* **54** (2025).
4. S. Markussen, K. Røed, "Inntektsulikhet og intergenerasjonell mobilitet [Income inequality and intergenerational mobility]" in Det norske samfunn [The Norwegian Society], I. Frønes, L. Kjølsrud, Eds. (Gyldendal Norsk Forlag, Oslo, 2022), chap. 33, pp. 383-408.
5. S. Markussen, K. Røed, Economic Mobility Under Pressure. *Journal of the European Economic Association* **18**, 1844-1885 (2019).
6. M. Bhuller, M. Mogstad, K. G. Salvanes, Life-Cycle Earnings, Education Premiums, and Internal Rates of Return. *Journal of Labor Economics* **35**, 993-1030 (2017).
7. L. Eika, L. J. Kirkebøen, Forskjeller i livsløpsinntekt mellom utdanningsgrupper [Differences in lifetime earnings between groups of education]. *Statistics Norway,* Technical Report (2023).
8. D. Card, The Causal Effect of Education on Earnings. *Handbook of Labor Economics* **3**, 1801-1863 (1999).
9. D. Card, Estimating the Return to Schooling: Progress on Some Persistent Econometric Problems. *Econometrica* **69**, 1127-1160 (2001).
10. Statistics Norway (2017) Norwegian Standard Classification of Education (https://www.ssb.no/en/utdanning/norwegian-standard-classification-of-education).
11. H. D. Zachrisson, E. Dearing, N. T. Borgen, A. M. J. Sandsør, L. A. Karoly, Universal Early Childhood Education and Care for Toddlers and Achievement Outcomes in Middle Childhood. *Journal of Research on Educational Effectiveness* **17**, 259-287 (2024).
12. A. Okbay *et al.*, Polygenic prediction of educational attainment within and between families from genome-wide association analyses in 3 million individuals. *Nature Genetics* **54**, 437-449 (2022).
13. M. Mills, N. Barban, F. Tropf, *An Introduction to Statistical Genetic Data Analysis.* MIT Press (2020).
14. G. Hemani *et al.*, The MR-Base platform supports systematic causal inference across the human phenome. *elife* **7**, e34408 (2018).
15. M. J. Moreira, Tests with correct size when instruments can be arbitrarily weak. *Journal of Econometrics* **152**, 131-140 (2009).
16. L. J. Howe *et al.*, Within-sibship genome-wide association analyses decrease bias in estimates of direct genetic effects. *Nature Genetics* **54**, 581-592 (2022).
17. A. Manichaikul *et al.*, Robust relationship inference in genome-wide association studies. *Bioinformatics* **26**, 2867-2873 (2010).
18. J. D. Angrist, J.-S. Pischke, *Mostly Harmless Econometrics: An Empiricist's Companion* (Princeton University Press, Princeton, 2009).
19. M. P. Keane, T. Neal, A Practical Guide to Weak Instruments. *Annual Review of Economics* **16** (2024).
20. T. W. Anderson, H. Rubin, Estimation of the Parameters of a Single Equation in a Complete System of Stochastic Equations. *The Annals of Mathematical Statistics* **20**, 46-63 (1949).
21. W. Zhang, C.-Y. Su, S. Yoshiji, T. Lu, MR Corge: sensitivity analysis of Mendelian randomization based on the core gene hypothesis for polygenic exposures. *Bioinformatics* **40**, btae666 (2024).

22. E. Chyn, B. Frandsen, E. C. Leslie. Examiner and Judge Designs in Economics: A Practitioner's Guide. *Journal of Economic Literature* **63** (2)**,** 401-39 (2025).
23. K. Imai, G. King, E. A. Stuart, Misunderstandings between experimentalists and observationalists about causal inference. *Journal of the Royal Statistical Society: Series A (Statistics in Society)* **171**, 481-502 (2008).
24. P. T. von Hippel, Multiply by 37 (or Divide by 0.027): A Surprisingly Accurate Rule of Thumb for Converting Effect Sizes From Standard Deviations to Percentile Points. *Educational Evaluation and Policy Analysis* **0**, 01623737241239677 (2024).
25. S. Burgess, S. G. Thompson, *Mendelian Randomization: Methods for Causal Inference Using Genetic Variants* (CRC Press, 2021).
26. E. Sanderson *et al.*, Mendelian randomization. *Nature Reviews Methods Primers* **2**, 6 (2022).
27. J. Bowden, G. Davey Smith, S. Burgess, Mendelian randomization with invalid instruments: effect estimation and bias detection through Egger regression. *International Journal of Epidemiology* **44**, 512-525 (2015).
28. J. Bowden, G. Davey Smith, P. C. Haycock, S. Burgess, Consistent Estimation in Mendelian Randomization with Some Invalid Instruments Using a Weighted Median Estimator. *Genetic Epidemiology* **40**, 304-314 (2016).
29. F. P. Hartwig, G. Davey Smith, J. Bowden, Robust inference in summary data Mendelian randomization via the zero modal pleiotropy assumption. *International Journal of Epidemiology* **46**, 1985-1998 (2017).
30. J. Zheng *et al.*, Recent Developments in Mendelian Randomization Studies. *Current Epidemiology Reports* **4**, 330-345 (2017).
31. T. G. Conley, C. B. Hansen, P. E. Rossi, Plausibly Exogenous. *Review of Economics and Statistics* **94**, 260-272 (2012).
32. F. Elwert, C. Winship, Endogenous Selection Bias: The Problem of Conditioning on a Collider Variable. *Annual Review of Sociology* **40**, 31-53 (2014).
33. N. Barban, E. De Cao, S. Oreffice, C. Quintana-Domeque, The effect of education on spousal education: A genetic approach. *Labour Economics* **71**, 102023 (2021).
34. D. Clarke, B. Matta, Practical considerations for questionable IVs. *The Stata Journal* **18**, 663-691 (2018).
35. S. Ishimaru, Empirical Decomposition of the IV-OLS Gap with Heterogeneous and Nonlinear Effects. *The Review of Economics and Statistics* **106**, 505-520 (2024).
36. N. M. Davies, M. Dickson, G. Davey Smith, F. Windmeijer, G. J. van den Berg, The causal effects of education on adult health, mortality and income: evidence from Mendelian randomization and the raising of the school leaving age. *International Journal of Epidemiology* 10.1093/ije/dyad104 (2023).
37. J. A. Mincer, "The Human Capital Earnings Function" in Schooling, Experience, and Earnings. (NBER, 1974), pp. 83-96.
38. J. D. Angrist, J.-S. Pischke, *Mastering 'Metrics: The Path from Cause to Effect* (Princeton University Press, Princeton, 2014).
39. C. Cinelli, A. Forney, J. Pearl, A crash course in good and bad controls. *Sociological Methods & Research* **53**, 1071-1104 (2024).
40. J. J. Heckman, J. E. Humphries, G. Veramendi, Returns to Education: The Causal Effects of Education on Earnings, Health, and Smoking. *Journal of Political Economy* **126**, S197-S246 (2018).
41. J. Heckman *et al.*, Earnings Functions and Rates of Return. *Journal of Human Capital* **2**, 1-31 (2008).
42. F. Buscha, M. Dickson, "Returns to Education: Individuals" in Handbook of Labor, Human Resources and Population Economics. (Springer, 2023), pp. 1-39.

43. R. Aaberge, M. Mogstad, V. Peragine, Measuring long-term inequality of opportunity. *Journal of Public Economics* **95**, 193-204 (2011).
44. StataCorp (2024) Stata Statistical Software: Release 18/MP (StataCorp LLC., College Station, TX).
45. S. Correia, REGHDFE: Stata module to perform linear or instrumental-variable regression absorbing any number of high-dimensional fixed effects. (2019).
46. S. Correia, IVREGHDFE: Stata module for extended instrumental variable regressions with multiple levels of fixed effects. (2018).
47. K. Finlay, L. Magnusson, M. Schaffer, WEAKIV: Stata module to perform weak-instrument-robust tests and confidence intervals for instrumental-variable (IV) estimation of linear, probit and tobit models. (2016).
48. R Core Team (2023) R: A language and environment for statistical computing. (R Foundation for Statistical Computing, Vienna, Austria).
49. S. Purcell *et al.*, PLINK: a tool set for whole-genome association and population-based linkage analyses. *The American Journal of Human Genetics* **81**, 559-575 (2007).
50. L. Jiang *et al.*, A resource-efficient tool for mixed model association analysis of large-scale data. *Nature Genetics* **51**, 1749-1755 (2019).

6. STROBE-MR checklist

STROBE-MR checklist of recommended items to address in reports of Mendelian randomization studies[1 2]

Item No.	Section	Checklist item	Page No.	Relevant text from manuscript
1	**TITLE and ABSTRACT**	Indicate Mendelian randomization (MR) as the study's design in the title and/or the abstract if that is a main purpose of the study	2	Genotyped data offer a new opportunity to address unobserved confounding and to estimate the returns to an additional year of schooling at any age by using quasi-randomly assigned genetic variants related to education as instrumental variables (Mendelian randomization (MR)).
	**INTRODUCTION**			
2	**Background**	Explain the scientific background and rationale for the reported study. What is the exposure? Is a potential causal relationship between exposure and outcome plausible? Justify why MR is a helpful method to address the study question	4-5	Two dominant empirical strategies have been used to address unobserved confounding: family-based designs and quasi-experiments (12). … Such designs tend to estimate lower returns than OLS but are still vulnerable to bias from unobserved confounding at the individual level (e.g., nonshared environments) (5, 12, 13, 15-17). … School reform-based studies evaluate specific reforms that raise schooling by one year at a particular age, however, which limits their generalizability to increases in schooling at other ages (5). … We address these limitations by … Mendelian randomization (MR), a form of IV analysis, leverages this random allocation as an exogenous shock to estimate the causal returns to schooling (22, 23).
3	**Objectives**	State specific objectives clearly, including pre-specified causal hypotheses (if any). State that MR is a method that, under specific assumptions, intends to estimate causal effects	5-6	… we estimate the economic returns to schooling with well-powered MR and sibling-MR using nationally representative Norwegian registry data and genetic data from the Norwegian Child, Mother, and Father Cohort

				Study (MoBa) (34). … To be a valid IV, the PIV^{EA} must (A1) be strongly associated with EA (relevance), (A2) share no common causes with the outcome (independence), (A3) only impact earnings through EA (exclusion), and (A4), under heterogeneous effects, only associate with EA in one direction (monotonicity) (22, 23, 26).
	**METHODS**			
4	**Study design and data sources**	Present key elements of the study design early in the article. Consider including a table listing sources of data for all phases of the study. For each data source contributing to the analysis, describe the following:		
	a)	Setting: Describe the study design and the underlying population, if possible. Describe the setting, locations, and relevant dates, including periods of recruitment, exposure, follow-up, and data collection, when available.	6	Our Norwegian setting is characterized by a small open economy, low economic inequality (45, 46), and a universal welfare state with practically free education. However, wealth inequality is comparatively high (47), child poverty and education gaps between rich and poor have increased (48, 49), and economic mobility among poor families has declined in recent decades (45), underscoring the need for stronger causal evidence about the role of education for earnings (50). … OLS (full population sample: N=1,255,604), sibling fixed-effects (sibling sample: N=966,976), twin fixed-effects (DZ) (DZ twin sample: N=3,219), twin fixed-effects (MZ) (MZ twin sample: N=2,630), MR (MoBa genotyped sample: N=109,800), sibling-MR (MoBa genotyped sibling sample: N=18,666), and Norway-only MR (MoBa genotyped estimation sample excluding individuals in the MoBa-within-sibling FGWAS discovery sample: N=89,179).
	b)	Participants: Give the eligibility criteria, and the sources and methods of selection of participants. Report the sample size, and whether any power or sample size calculations were carried out prior to the main analysis	SI p. 2	Norwegian country of birth and at least one parent Norway as country of birth; other parent restricted to Europe due to European genetic ancestry data; Birth cohorts 1959−1982; Registered mother and father, education, earnings when aged 34−40.

		c)	Describe measurement, quality control and selection of genetic variants	SI p.4-5	We constructed PIV^{EA} using weights and direction of effects of variants identified in the EA4 GWAS (12), excluding 23andMe and MoBa participants. We excluded SNPs not available in MoBa and the 1000 Genome Project reference set. Using Plink 1.9, we then identified variants independently associated with EA, with a clumping threshold of r^2<0.001, LD=10,000 *kb* at *p*<5.0×10–8. This left 335 SNPs associated with EA, which we used to construct the PIV^{EA} for each individual participant, with Plink 1.9. Strict clumping threshold of r^2<0.001 follows the current two-sample MR R package recommendation (14).
		d)	For each exposure, outcome, and other relevant variables, describe methods of assessment and diagnostic criteria for diseases	SI section 1.3	Earnings and education described in detail in SI Appendix section 1.3; diagnostic criteria for disease not relevant.
		e)	Provide details of ethics committee approval and participant informed consent, if relevant	20	MoBa is a population-based pregnancy cohort study conducted by the Norwegian Institute of Public Health. Participants were recruited from all over Norway from 1999-2008. MoBa is based on informed consent from all participants in accordance with Norwegian regulations on population-based health surveys (80). The women consented to participation in 41% of the pregnancies. In 87.3% of participating pregnancies, fathers were also invited to participate, with 82.9% providing consent (74). The cohort includes approximately 114,500 children, 95,200 mothers and 75,200 fathers. MoBa is regulated by the Norwegian Health Registry Act. The current study was approved by The Regional Committees for Medical and Health Research Ethics (2017/2205).
5	**Assumptions**		Explicitly state the three core IV assumptions for the main analysis (relevance, independence and exclusion restriction) as well assumptions for any additional or sensitivity analysis	6	To be a valid IV, the PIV^{EA} must (A1) be strongly associated with EA (relevance), (A2) share no common causes with the outcome (independence), (A3) only impact earnings through EA (exclusion), and (A4), under

				heterogeneous effects, only associate with EA in one direction (monotonicity) (22, 23, 26).
6	**Statistical methods: main analysis**	Describe statistical methods and statistics used		
	a)	Describe how quantitative variables were handled in the analyses (i.e., scale, units, model)	9 & SI sections 1.3 and 2	Labor market earnings were the mean of the top-three earnings years between ages 34 and 40. Years of schooling were the highest educational attainment by age 33. All analyses were adjusted for covariates, including sex, parents' income and education, number of children in the family, birth year, birth order, and parents' age at birth (sibling and twin fixed-effects models and sibling-MR do not adjust for covariates for shared family characteristics that are constant between siblings) (SI Appendix sections 1.3 and 2).
	b)	Describe how genetic variants were handled in the analyses and, if applicable, how their weights were selected	SI p.4-5	We constructed PIV^{EA} using weights and direction of effects of variants identified in the EA4 GWAS (12), excluding 23andMe and MoBa participants. We excluded SNPs not available in MoBa. Using Plink 1.9, we then identified variants independently associated with EA, with a clumping threshold of r^2<0.001, LD=10,000 *kb* at p<5.0 × 10–8. This left 335 SNPs associated with EA, which we used to construct the PIV^{EA} for each individual participant, with Plink 1.9. Strict clumping threshold of r^2<0.001 follows the current two-sample MR R package recommendation (14).
	c)	Describe the MR estimator (e.g. two-stage least squares, Wald ratio) and related statistics. Detail the included covariates and, in case of two-sample MR, whether the same covariate set was used for adjustment in the two samples	9, 21, SI sections 1.3 and 2	… we estimated MR using 2SLS (p.21); All analyses were adjusted for covariates, including sex, parents' income and education, number of children in the family, birth year, birth order, and parents' age at birth (sibling and twin fixed-effects models and sibling-MR do not adjust for covariates for shared family characteristics) (SI Appendix sections 1.3 and 2) (p.9).

		d)	Explain how missing data were addressed	SI p.7	For all models, we report models with and without covariate adjustment. Adjusted analyses were conducted on complete cases with nearly no missing data (<1%). Standard errors are clustered by family and birth cohort in family (sibling and twin) models, and by birth cohort otherwise. For MR, we report Anderson-Rubin (AR) tests and 95% confidence intervals (20).
		e)	If applicable, indicate how multiple testing was addressed	-	NA
7	**Assessment of assumptions**		Describe any methods or prior knowledge used to assess the assumptions or justify their validity	5-6	Third, we conduct extensive analyses to assess the key assumptions underlying MR. To be a valid IV, the PIV^{EA} must (A1) be strongly associated with EA (relevance), (A2) share no common causes with the outcome (independence), (A3) only impact earnings through EA (exclusion), and (A4), under heterogeneous effects, only associate with EA in one direction (monotonicity) (22, 23, 26). We test the relevance assumption (A1) using weak-instrument diagnostics (37). We address possible violations of the independence assumption (A2) (e.g., due to population stratification, assortative mating, or dynastic effects), first, by adjusting for key covariates (e.g., parental education and earnings) in MR analyses in our full population sample; second, by demonstrating balance on observed covariates (38); and, third, by estimating well-powered sibling-MR that adjusts for family-level unobserved confounding (22, 25, 28, 29). We address possible violations of the exclusion assumption (A3) (e.g., due to pleiotropic direct effects, whereby the genetic variants in the PIV^{EA} might impact earnings directly through pathways other than EA) in multiple ways. First, all of our MR analyses use only strongly associated genetic variants to maximize relevance and minimize potential pleiotropic variants (39, 40); second, we test for pleiotropy

				using an MR-Egger intercept test (23); third, we employ multiple pleiotropy-robust estimators, including MR-Egger, MR-Median, MR-Mode, and MR-Corge (22, 23, 40); fourth, we conduct a formal sensitivity analysis that examines the robustness of our estimates to a range of potential pleiotropic direct effects (41). We assess monotonicity (A4) by inspecting covariate-specific weights in the MR-OLS decomposition (36).
8	**Sensitivity analyses and additional analyses**	Describe any sensitivity analyses or additional analyses performed (e.g. comparison of effect estimates from different approaches, independent replication, bias analytic techniques, validation of instruments, simulations)	5	Second, we triangulate estimates for the returns to schooling across multiple identification strategies that rely on different assumptions: OLS with covariate-adjustment, family-based designs with sibling and twin fixed-effects, and MR. Collectively, these results provide insights into potential bias sources (35). We elucidate differences in the returns to schooling estimated by OLS and MR using a novel MR-OLS decomposition method (36).
9	**Software and pre-registration**			
	a)	Name statistical software and package(s), including version and settings used	SI section 4	Data preparation, individual-level analyses, and data visualization was conducted in Stata MP 18 (46), using reghdfe (47) and ivreghdfe (48), plausexog (33) for the UCI sensitivity analysis, ivolsdec (37) for MR-OLS decomposition, and weakiv for AR-test and 95% confidence intervals (49). Two-sample MR data preparation, analyses, and data visualization were conducted in R v. 4.2.3 (50), using the TwoSampleMR package (14). Plink 1.9 (51) was used to create PIV^{EA} and fastGWA (52) was used to obtain SNP-earnings effects.
	b)	State whether the study protocol and details were pre-registered (as well as when and where)	-	NA

	**RESULTS**			
10	**Descriptive data**			
	a)	Report the numbers of individuals at each stage of included studies and reasons for exclusion. Consider use of a flow diagram	SI p.2	Figure S1. Flowchart for sample construction.
	b)	Report summary statistics for phenotypic exposure(s), outcome(s), and other relevant variables (e.g. means, SDs, proportions)	SI p.8	Table S1. Summary statistics for population samples and genotyped MoBa samples.
	c)	If the data sources include meta-analyses of previous studies, provide the assessments of heterogeneity across these studies	-	NA
	d)	For two-sample MR:<br>i. Provide justification of the similarity of the genetic variant-exposure associations between the exposure and outcome samples<br>ii. Provide information on the number of individuals who overlap between the exposure and outcome studies	21, SI section 1.3	We constructed a PIV^{EA} using weights and direction of effects of variants identified in the EA4 GWAS (24), excluding 23andMe and MoBa participants. We excluded SNPs not available in MoBa and the 1000 Genome Project reference set. Using Plink 1.9, we then identified variants independently associated with EA, with a strict clumping threshold of r^2<0.001, LD=10,000 *kb*, at *p*<5.0×10–8 following recommendations for MR-analyses (40). This left 335 SNPs associated with EA4 that we used to construct the allele scores for individual participants (SI Appendix, section 1.3). … We use the same 335 SNPs in our summary-level MR analyses.
11	**Main results**			
	a)	Report the associations between genetic variant and exposure, and between genetic variant and outcome, preferably on an interpretable scale	SI p.20	Figure S6. Two-sample Mendelian randomization scatterplot with pleiotropy-robust estimators. SNP-schooling (*x*-axis) and SNP-earnings (*y*-axis) associations and 95% confidence intervals plotted per SNP. Lines represent fitted values from the estimators inverse-variance weighted (IVW-MR), MR-Egger, MR-Median (weighted median), and MR-Mode (weighted mode and simple mode).

		b)	Report MR estimates of the relationship between exposure and outcome, and the measures of uncertainty from the MR analysis, on an interpretable scale, such as odds ratio or relative risk per SD difference	SI p.2 and p.18	Table S2. Returns to schooling estimates (p.9) and Table S4. Returns to schooling estimates from pleiotropy-robust two-sample Mendelian randomization estimators. Standard error in parentheses (p.18).
		c)	If relevant, consider translating estimates of relative risk into absolute risk for a meaningful time period	-	NA
		d)	Consider plots to visualize results (e.g. forest plot, scatterplot of associations between genetic variants and outcome versus between genetic variants and exposure)	SI p.20	Figure S6. Two-sample Mendelian randomization scatterplot with pleiotropy-robust estimators. SNP-schooling (*x*-axis) and SNP-earnings (*y*-axis) associations and 95% confidence intervals plotted per SNP. Lines represent fitted values from the estimators inverse-variance weighted (IVW-MR), MR-Egger, MR-Median (weighted median), and MR-Mode (weighted mode and simple mode).
12	**Assessment of assumptions**				
		a)	Report the assessment of the validity of the assumptions	SI section 3.3	Full section: Assessing MR assumptions
		b)	Report any additional statistics (e.g., assessments of heterogeneity across genetic variants, such as I^2, Q statistic or E-value)	SI p.15-16	Cochran's Q-statistic (Q=476, *df*=334, $p<0.001$) indicates heterogeneity across SNP-specific estimates for the returns to schooling. Under the additional assumption of constant returns to schooling across the population (no effect heterogeneity), this could indicate horizontal (but not necessarily biasing directional) pleiotropy; alternatively, it could indicate effect heterogeneity in the returns to schooling.

13	**Sensitivity analyses and additional analyses**				
		a)	Report any sensitivity analyses to assess the robustness of the main results to violations of the assumptions	SI section 3.3	Full section: Assessing MR assumptions
		b)	Report results from other sensitivity analyses or additional analyses	SI section 3.3	Full section: Assessing MR assumptions
		c)	Report any assessment of direction of causal relationship (e.g., bidirectional MR)		NA
		d)	When relevant, report and compare with estimates from non-MR analyses	8	Figure 2. Returns to schooling estimates from OLS, sibling fixed-effects, twin fixed-effects, MR, and sibling-MR models.
		e)	Consider additional plots to visualize results (e.g., leave-one-out analyses)	SI p.19	Figure S5. Leave-one-out sensitivity analysis for MR-Egger.
	**DISCUSSION**				
14	**Key results**		Summarize key results with reference to study objectives	15	We estimated the economic returns to schooling from population-wide registry and genetic data across multiple, increasingly rigorous statistical approaches. Estimates were smallest in fixed-effects models, larger in OLS, and largest in MR. Our key results were remarkably consistent across designs in three respects. First, prime-age returns to schooling (ranging from 3.2% to 9.7% for an additional year) were positive, statistically significant, and large (Figure 2, Table S2) (51). Second, life-cycle analyses showed that returns to schooling varied by age. Returns were negative at young ages due to forgone labor market experience and earnings, became positive around age 27, and kept growing until the end of workers' careers (Figure 3, Table S9). Third, the lifetime returns to schooling as measured by the IRR (ranging from 6.8% to 10.1%, Table S9) exceeded opportunity costs as proxied by the market interest rate (2.3%). Regardless of the

					estimation strategy, the evidence showed that education pays off.
15	**Limitations**		Discuss limitations of the study, taking into account the validity of the IV assumptions, other sources of potential bias, and imprecision. Discuss both direction and magnitude of any potential bias and any efforts to address them	18-19	Full section: Limitations of MR
16	**Interpretation**				
		a)	Meaning: Give a cautious overall interpretation of results in the context of their limitations and in comparison with other studies	19	Our MR approach exemplifies insights gained from combining genetic data with quasi-experimental designs. By exploiting the genetic lottery at birth as an instrument, we estimate that one additional year of schooling results in 8% higher annual earnings in our main MR model. While conventional approaches yield lower estimates, all exceed opportunity costs of education as proxied by the market interest rate. These findings reinforce confidence in the economic value of education, with important implications for individual educational decisions (1) and policy development (2).
		b)	Mechanism: Discuss underlying biological mechanisms that could drive a potential causal relationship between the investigated exposure and the outcome, and whether the gene-environment equivalence assumption is reasonable. Use causal language carefully, clarifying that IV estimates may provide causal effects only under certain assumptions	16	While researchers can never verify that all necessary assumptions hold (26, 27), our MR results held up to a comprehensive battery of tests, falsification tests, robustness checks, and a formal sensitivity analysis (SI Appendix, section 3.3), suggesting that MR is valid for identifying the causal returns to schooling. (p.16) … Our finding that MR estimates are higher than our fixed-effects and OLS estimates (Figure 2) replicates the well-known pattern that IV (including MR) estimates for the returns to schooling exceed more conventional estimates (28, 29, 63) (p.16-17).
		c)	Clinical relevance: Discuss whether the results have clinical or public policy relevance, and to what extent they inform effect sizes of possible interventions	19	Rising educational attainment and technological changes have intensified debates about the economic value of education (76, 77). This study advances the returns to schooling literature by demonstrating how the careful integration of genetic data can help investigate

				hard-to-answer causal questions in the social sciences. Our MR approach exemplifies insights gained from combining genetic data with quasi-experimental designs. By exploiting the genetic lottery at birth as an instrument, we estimate that one additional year of schooling results in 8% higher annual earnings in our main MR model. While conventional approaches yield lower estimates, all IRRs for all approaches exceed opportunity costs of education as proxied by the market interest rate. These findings reinforce confidence in the economic value of education, with important implications for individual educational decisions (1) and policy development (2).
17	**Generalizability**	Discuss the generalizability of the study results (a) to other populations, (b) across other exposure periods/timings, and (c) across other levels of exposure	19	Finally, the generalizability of our MR estimates may be limited in three respects. First, our MR models analyze the MoBa cohort study, whose participants are healthier and more socioeconomically advantaged than the average Norwegian (73). However, prior work supports the generalizability of associations from MoBa to the general population (34, 74, 75). Second, our study was conducted in the Norwegian universal welfare state with nearly free education and other policies aimed at reducing barriers to education. Therefore, our findings may not generalize to other institutional and national contexts. Third, gains to schooling may vary across individuals in ways not captured in our heterogeneity analysis (SI Appendix, Table S8), e.g., by fields of study (68).
	**OTHER INFORMATION**			
18	**Funding**	Describe sources of funding and the role of funders in the present study and, if applicable, sources of funding for the databases and original study or studies on which the present study is based	23	TW, HZ, NTB, and EY were partly supported by the Research Council of Norway (RCN) through its Centres of Excellence scheme (grant number 331640). TW, HZ, PAD, and EY are supported by the European Research Council

				(grant number 101045526). EY is supported by the European Research Council (ERC) (grant number 101073237), The Swedish Research Council (grant number 2024-06499 VR), and RCN (grant numbers 336078, 288083). TW and HZ were supported by funding from the ERC Consolidator (grant number 818425). NTB was supported by funding from the ERC (grant number 101115949). FE was supported by a grant from the Eunice Kennedy Shriver National Institute of Child Health and Human Development (grant number P2C HD047873).
19	**Data and data sharing**	Provide the data used to perform all analyses or report where and how the data can be accessed, and reference these sources in the article. Provide the statistical code needed to reproduce the results in the article, or report whether the code is publicly accessible and if so, where	23	Code is available on GitHub (https://github.com/tarjeiw/mr-iv-returns). … Data availability for research purposes is subject to strict Norwegian privacy regulations. Details on access to microdata can be obtained from mikrodata@ssb.no and datatilgang@fhi.no.
20	**Conflicts of Interest**	All authors should declare all potential conflicts of interest	1	The authors declare no conflict of interest.

This checklist is copyrighted by the Equator Network under the Creative Commons Attribution 3.0 Unported (CC BY 3.0) license.

1. Skrivankova VW, Richmond RC, Woolf BAR, Yarmolinsky J, Davies NM, Swanson SA, et al. Strengthening the Reporting of Observational Studies in Epidemiology using Mendelian Randomization (STROBE-MR) Statement. JAMA. 2021;under review.

2. Skrivankova VW, Richmond RC, Woolf BAR, Davies NM, Swanson SA, VanderWeele TJ, et al. Strengthening the Reporting of Observational Studies in Epidemiology using Mendelian Randomisation (STROBE-MR): Explanation and Elaboration. BMJ. 2021;375:n2233.
